# CK1δ stimulates ubiquitination‐dependent proteasomal degradation of ATF4 to promote chemoresistance in gastric Cancer

**DOI:** 10.1002/ctm2.587

**Published:** 2021-10-14

**Authors:** Lifeng Feng, Muchun Li, Xinyang Hu, Yiling Li, Liyuan Zhu, Miaoqin Chen, Qi Wei, Wenxia Xu, Qiyin Zhou, Weikai Wang, Dingwei Chen, Xian Wang, Hongchuan Jin

**Affiliations:** ^1^ Laboratory of Cancer Biology Key Lab of Biotherapy in Zhejiang Cancer Center of Zhejiang University Sir Run Run Shaw Hospital Medical School of Zhejiang University Hangzhou China; ^2^ Central Laboratory Affiliated Jinhua Hospital Medical School of Zhejiang University Jinhua Zhejiang China; ^3^ Department of General Surgery Sir Run Run Shaw Hospital Medical School of Zhejiang University Hangzhou China; ^4^ Department of Medical Oncology Sir Run Run Shaw Hospital Medical School of Zhejiang University Hangzhou China

**Keywords:** ATF4, chemoresistance, CK1δ, gastric cancer, phosphorylation, ubiquitination

## Abstract

Chemoresistance remains a major obstacle to successful cancer therapy, especially for advanced cancers. It used to be recognised as a stable outcome resulting from genetic changes. However, recent studies showed that chemoresistance can also be unstable and reversible with the involvement of non‐genetic alterations. In the present study, we found that activating transcription factor 4 (ATF4) is downregulated in chemoresistant gastric cancer cells. The over‐expression of ATF4 reversed chemoresistance by activating CHOP transcription to enhance drug‐induced apoptosis, and vice versa. Moreover, casein kinase 1 delta (CK1δ) was identified as the kinase responsible for ATF4‐S219 phosphorylation, which triggered βTrCP‐mediated ATF4 polyubiquitination to promote its proteasomal degradation subsequently. Interestingly, drug withdrawal gradually restored chemosensitivity as well as ATF4 expression in chemoresistant cells, highlighting the dependence of dynamic drug resistance on ATF4 protein expression. In line with these findings, the inhibition of ATF4 protein degradation by CK1δ or proteasome inhibitors overcame chemoresistance both in vitro and in vivo. Taken together, these results indicate that CK1δ stimulates βTrCP‐dependent ATF4 polyubiquitination and subsequent proteasomal degradation to promote chemoresistance in gastric cancer. Stabilisation of the ATF4 protein with bortezomib (BTZ), an anticancer drug that inhibits proteasomal degradation, might be a rational strategy to improve chemotherapeutic efficacy in gastric cancer.

## Graphical abstract

Chemoresistance could be unstable and reversible. ATF4 protein level is negatively correlated to chemoresistance in gastric cancer. CK1δ stimulates ATF4 poly‐ubiquitinated degradation is responsible for the dynamic chemoresistance. Targeting ATF4 could be a rational strategy to reverse chemoresistance in gastric cancer.

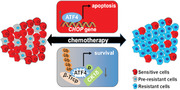

## 1 INTRODUCTION

Chemotherapy is still the main clinical treatment for many cancers, especially for advanced cancers. Unfortunately, resistance to chemotherapy (chemoresistance) will inevitably come up for most patients. Chemoresistance has been the primary cause of clinical failure and leads to the poor overall survival of advanced cancer patients.^1^, ^2^ Therefore, it is fundamental to design new approaches to overcome chemoresistance and win the war on cancer.

During chemoresistance development, multiple signalling pathways including drug uptake and efflux, DNA damage repair, metabolism, as well as apoptosis induction are reprogrammed.^3^ Genome instability is believed to promote cellular heterogeneity,^4^ thus providing a strong advantage for cancer cells to develop chemoresistance via exposure selection of drug‐resistant clones.^5^ On the other hand, recent studies have indicated that unlike genetic alterations, inheritable epigenetic changes contribute to fluctuating drug tolerance. It has been observed that some chemoresistant cancer patients become re‐sensitive to the same drug after a ‘drug holiday’.^6^, ^7^ For example, a proportion of relapsed breast cancer patients respond to the same drugs that they have responded initially.^8^, ^9^ Meanwhile, non‐small cell lung cancer (NSCLC) patients could restore response to gefitinib after stopping the drug for a period of time because of acquired resistance.^10^, ^11^ According to above findings, intermittent chemotherapy appears to be more effective in some cancers.

Despite rapid progress in precise oncology, cisplatin (DDP) containing regimen is still one of the dominant chemotherapeutics, especially for cancers with limited targeted therapy options, such as gastric cancer. Multiple mechanisms, including upregulated p‐glycoprotein expression,^12^ an enhanced DNA damage response^13^, ^14^ and increased glycolysis,^15^ have been reported in cancer cells resistant to cisplatin. Moreover, as described above, platinum agents could become effective again for relapsed ovarian cancer patients resistant to platinum‐based therapy.^16^ However, the underlying mechanisms of how patients become re‐sensitive to these agents remain largely undefined.

Increasing studies show that activating transcription factor 4 (ATF4) is involved in the development of chemoresistance of many cancers. ATF4 is a stress response protein upregulated under various stress conditions, including endoplasmic reticulum (ER) stress and amino acid depletion. It activates downstream genes involved in antioxidant response, as well as unfolded protein response.^17^, ^18^ Upregulated ATF4 expression contributes to drug resistance by transcriptionally activating pro‐survival pathways, including redox homeostasis and autophagy.^19^, ^20^ Interestingly, ATF4 could also activate the transcription of certain pro‐apoptotic genes, such as C/EBP homologous protein 10 (CHOP, also known as DDIT3), to induce apoptotic cell death.^21^, ^22^ Thus, the role of ATF4 in chemoresistance is complicated and needs context‐dependent investigations. In addition, ATF4 protein expression is mainly regulated by eukaryotic initiation factor 2 alpha (eIF2α) phosphorylation‐dependent translation and ubiquitination‐dependent proteasomal degradation.^17^, ^23^, ^24^ While several kinases for eIF2α phosphorylation have been identified, the protein kinases responsible for the serine 219 (S219) phosphorylation of ATF4, which is critical for its polyubiquitination‐dependent proteasomal degradation,^23^ is still unknown.

In the present study, we reported that ATF4/CHOP axis is downregulated to inhibit drug‐induced apoptosis in chemoresistant gastric cancer. Protein kinase casein kinase 1 delta (CK1δ) phosphorylates ATF4 at S219 to promote SCF‐βTrCP‐mediated polyubiquitination and subsequent proteasomal degradation of ATF4. This dynamic regulation of ATF4 protein abundance is critical to chemosensitivity. Restoration of ATF4 protein expression by CK1δ or proteasome inhibitor effectively overcame chemoresistance by enhancing apoptosis in gastric cancer cells both in vitro and in vivo.

**HIGHLIGHTS**

1. Chemoresistance could be unstable and reversible.
2. Downregulation of ATF4/CHOP axis contributes to the chemoresistance in gastric cancer.
3. CK1δ stimulated polyubiquitination‐dependent proteasomal degradation of ATF4 is responsible for the dynamic chemoresistance in gastric cancer.
4. Stabilising ATF4 could be a rational strategy to reverse chemoresistance.

## 2 RESULTS

### 2.1 ATF4 is downregulated to promote chemoresistance in gastric cancer

**FIGURE 1:**
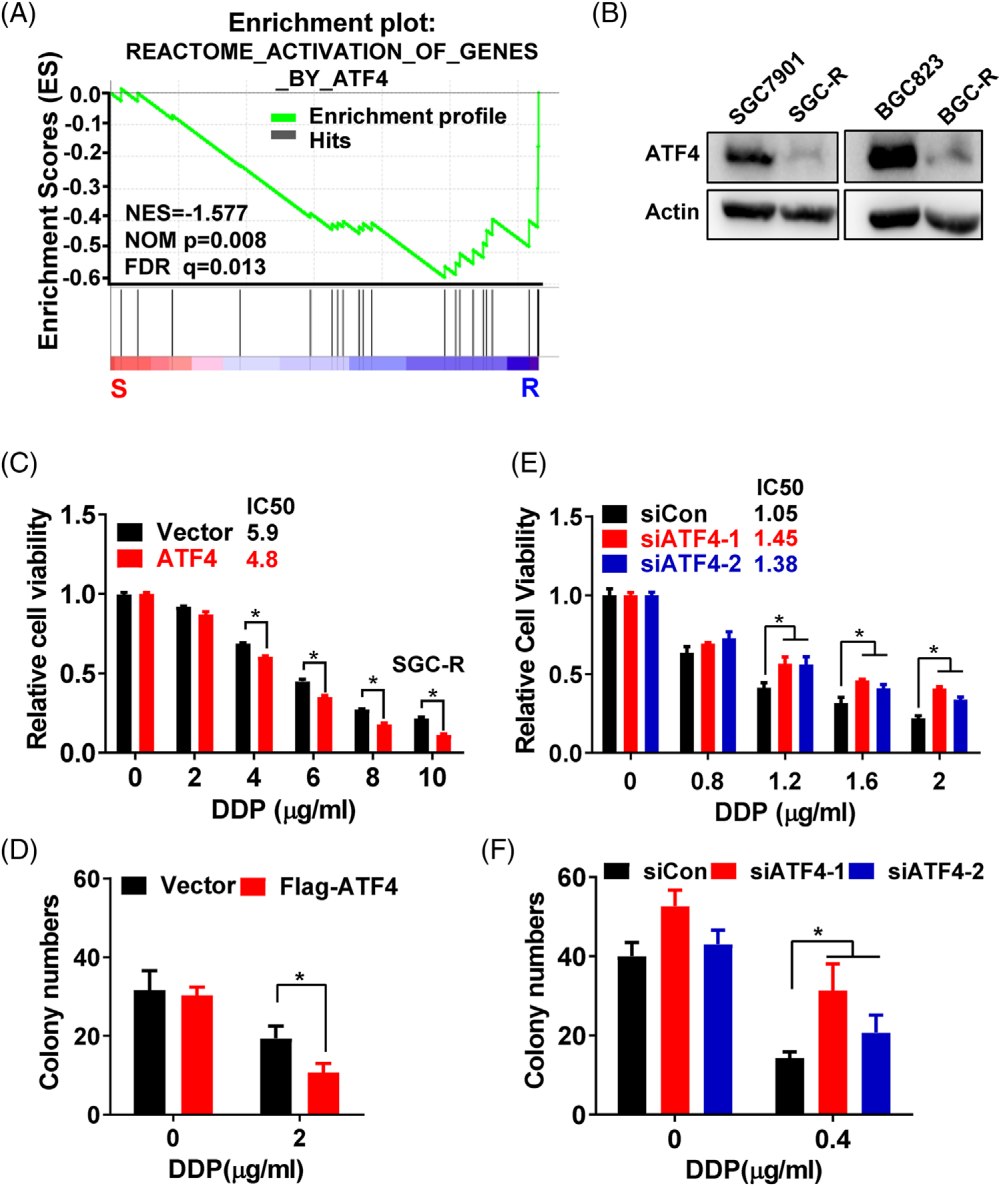
ATF4 is downregulated to promote chemoresistance in gastric cancer. (A) The gene expression profiles were analysed by Gene Set Enrichment Analysis (GSEA) in chemoresistant cells (SGC‐R and BGC‐R) and their parental sensitive cells (SGC7901 and BGC823), assessing the correlation to ATF4 target gene signatures in resistant cells compared to sensitive cells, ‘R’ represent drug‐resistant cells, ‘S’ represent sensitive cells. (B) Expression of ATF4 in chemoresistant cells (SGC‐R and BGC‐R) and their parental sensitive cells (SGC7901 and BGC823) was detected by western blotting, and Actin was used as loading control. (C) Cell viability of SGC‐R cells with ATF4 over‐expression, and DDP treatment for 24 h was measured by MTS assay. (D) The clonal growth of SGC‐R cells with ATF4 over‐expression, and DDP treatment for 7 days was assayed in Matrigel. (E) Cell viability of SGC7901 cells with ATF4 knockdown, and DDP treatment for 24 h was measured by MTS assay. (F) The clonal growth of SGC7901 cells with ATF4 knockdown, and DDP treatment for 7 days was assayed in Matrigel

In an effort to clarify the mechanisms underlying chemoresistance of gastric cancer, gene set enrichment analysis (GSEA) (http://www.broadinstitute.org/gsea/) was performed to analyse the global gene expression profiles in our previously established chemoresistant gastric cancer cells (SGC‐R and BGC‐R), as well as in their parental chemosensitive cells (SGC7901 and BGC823), respectively.^25^ GESA showed that the ATF4 target gene signatures were negatively enriched in chemoresistant cells compared to sensitive cells (Figures 1A and S1A). It means that the expression of ATF4 downstream target genes was downregulated in chemoresistant cells, indicating the relevance of ATF4 inactivation to chemoresistance. Indeed, compared to sensitive cells, ATF4 was downregulated in chemoresistant cells (Figure 1B). Meanwhile, we determined ATF4 expression in other gastric cancer cell lines, and measured the cell viability inhibition IC50 of each cell lines to cisplatin (DDP) by MTS assay (Figure S1B, C). It was found that the viability inhibition of gastric cancer cells by DDP was correlated to ATF4 expression levels. In addition, over‐expression of exogenous ATF4 in resistant cells evidently potentiated viability inhibition (low IC50) and retarded clonal growth under DDP treatment compared to vector group (Figures 1C, D and S1D–F), which means that over‐expressed ATF4 in resistant cells reversed the resistance to DDP. On the other hand, ATF4 knockdown in sensitive cells significantly increased the IC50 and promoted clonal growth under DDP treatment compared to control group, revealing that knockdown ATF4 expression in sensitive cells resulted in chemoresistance (Figures 1E, F and S1G–I). Taken together, these results indicated that ATF4 plays a critical role in repressing chemoresistance in gastric cancer cells.

### 2.2 ATF4 stimulates drug‐induced apoptosis

**FIGURE 2:**
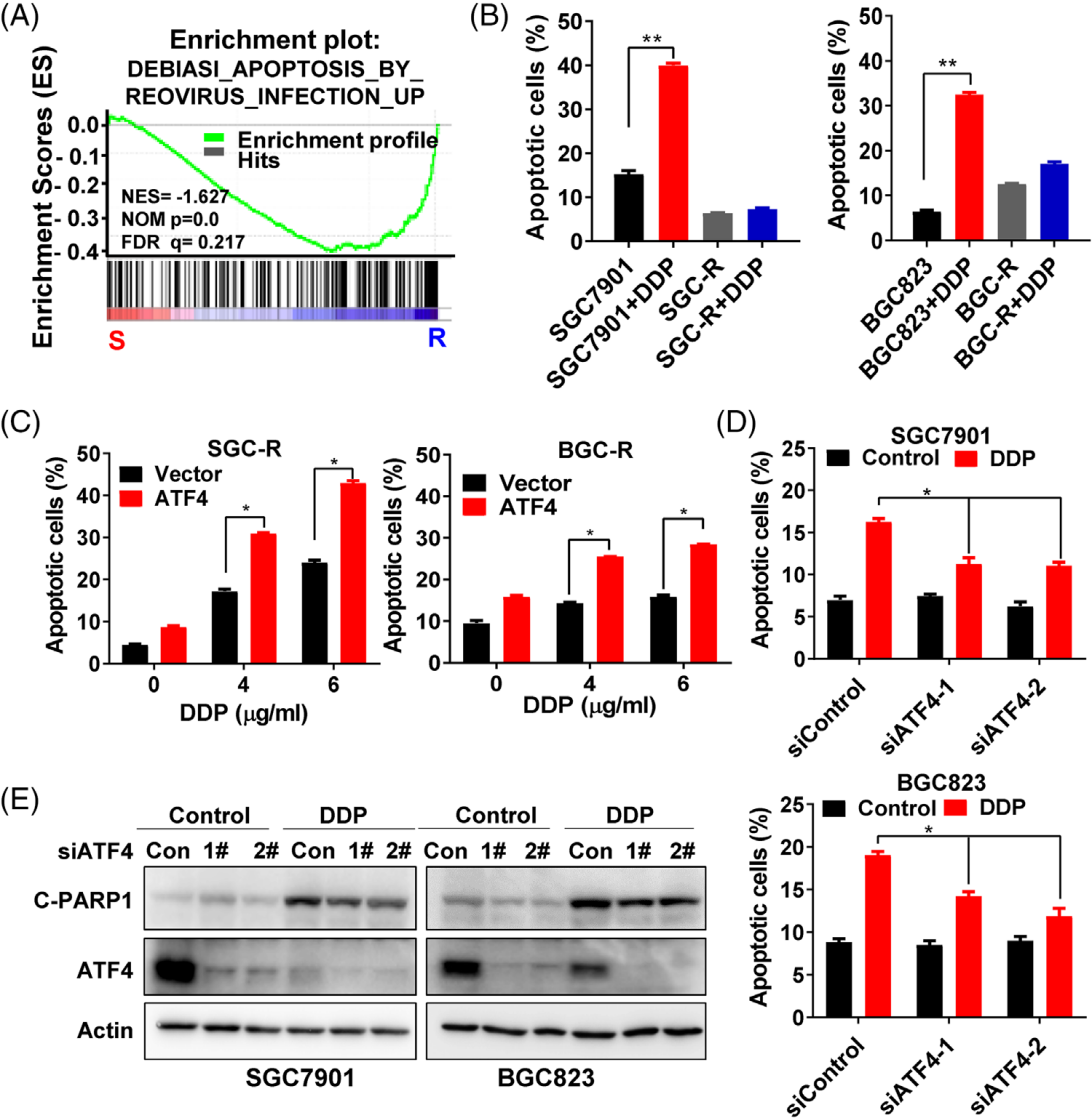
ATF4 stimulates drug‐induced apoptosis. (A) To assess the correlation of the expression of apoptosis‐related gene signature in resistant cells compared to sensitive cells, GSEA was used to analyse the gene expression profiles in chemoresistant cells (SGC‐R and BGC‐R) and their parental sensitive cells (SGC7901 and BGC823). (B) Apoptosis of SGC7901 and SGC‐R (left) or BGC823 and BGC‐R (right) under DDP (1.2 μg/ml) incubation for 24 h was measured by a PI/Annexin V double staining assay. (C) Apoptosis of SGC‐R (left) or BGC‐R (right) cells with ATF4 over‐expression and DDP treatment for 24 h was measured. (D) Apoptosis of SGC7901 (up) or BGC823 (down) cells with ATF4 knockdown and DDP treatment for 24 h was measured by a PI/Annexin V double staining assay. (E) The expression of apoptosis marker cleave‐PARP1 (C‐PARP1) and ATF4 in SGC7901 (left) or BGC823 (right) cells with ATF4 knockdown and DDP (1.2 μg/ml) treatment for 24 h was detected by western blotting

To evaluate whether apoptosis activation was implicated in chemoresistance, a common apoptosis‐related gene signature–‘DEBIASI_ Apoptosis_By_Reovirus’ selected from GSEA online database (http://www.gsea‐msigdb.org/gsea/login.jsp) was used for GSEA. The result showed that expression of apoptotic signalling genes was negatively enriched in chemoresistant cells compared to sensitive cells (Figure 2A), indicating that apoptosis might be inhibited in resistant cells. Previous studies have found that ATF4 is implicated in the regulation of apoptosis,^26^ which prompted us to further investigate the role of ATF4 in drug‐induced apoptosis. First, we confirmed that DDP‐induced significant apoptosis in chemosensitive cells but not in chemoresistant cells (Figures 2B and S2A). In addition, over‐expression of ATF4 in chemoresistant cells markedly increased the number of apoptotic cells (Figures 2C and S2B), and the level of apoptosis marker cleaved‐PARP1 (C‐PARP1) after DDP treatment (Figure S2C), confirming that over‐expressed ATF4 reversed chemoresistance by enhancing DDP‐induced apoptosis. On the other hand, knockdown of ATF4 in sensitive cells dramatically decreased DDP‐induced apoptotic cells (Figures 2D and S2D), and C‐PARP1 level (Figure 2E), indicating that ATF4 knockdown contributed to chemoresistance by attenuating DDP‐induced apoptosis. These data suggested that downregulation of ATF4 contributes to chemoresistance, at least partially, by inhibiting drug‐induced apoptosis.

### 2.3 ATF4 stimulates drug‐induced apoptosis by activating CHOP transcription

**FIGURE 3:**
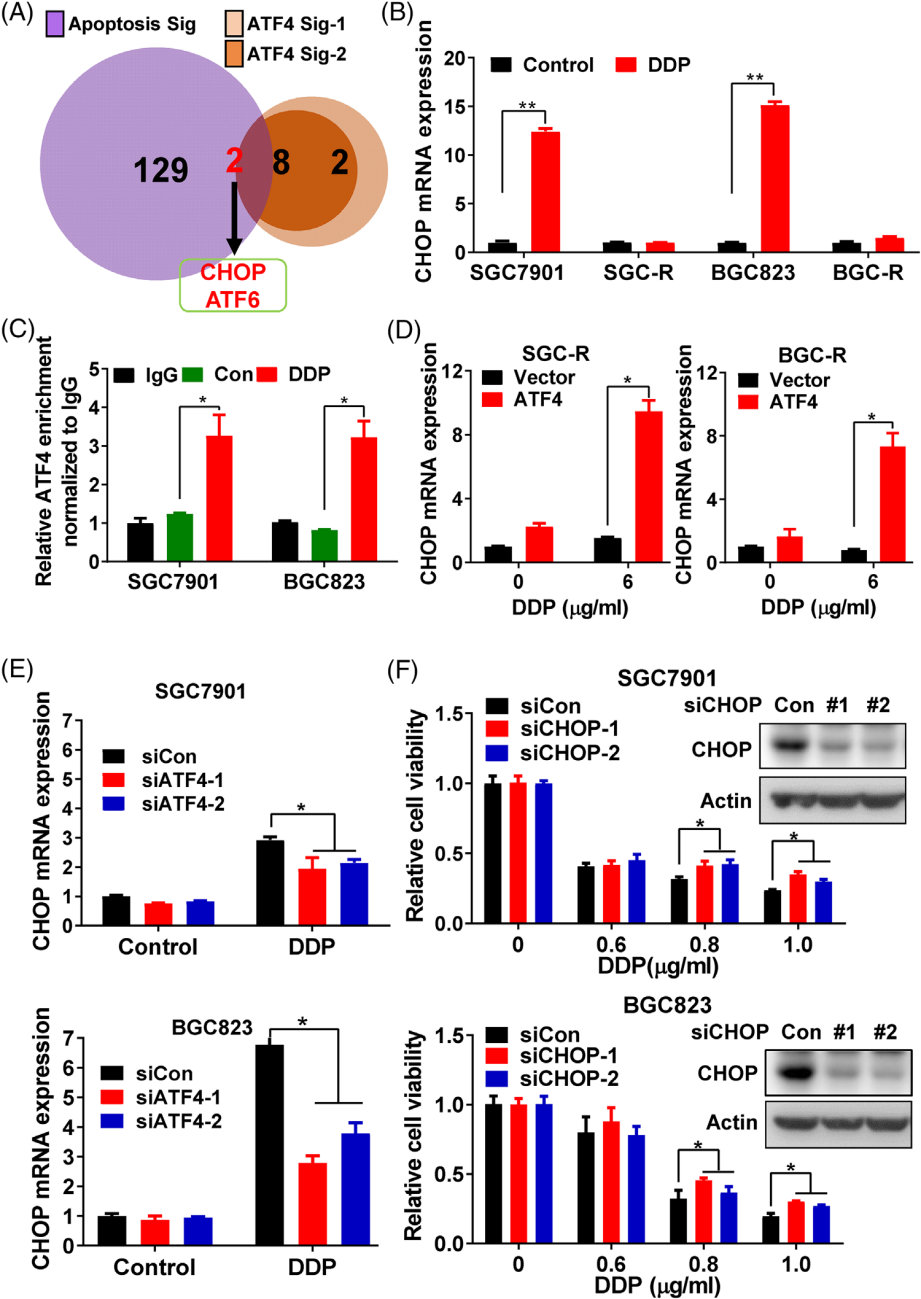
ATF4 stimulates drug‐induced apoptosis by activating CHOP transcription. (A) The GSEA‐enriched ATF4 target genes from the two signatures (ATF4 sig‐1 and ATF4 sig‐2) was intersected with enriched apoptosis related genes from apoptosis signature (Apoptosis sig) to identify the implicated apoptosis related genes. (B) CHOP mRNA expression in sensitive cells (SGC7901 and BGC823) or resistant cells (SGC‐R and BGC‐R) under DDP (1.2 μg/ml) incubation for 24 h was measured by qRT‐PCR. (C) CHIP was performed to detect the binding of ATF4 to CHOP promoter in SGC7901 or BGC823 cells with DDP (1.2 μg/ml) treatment for 6 h, rabbit IgG was used as control. (D) After ATF4 over‐expression and DDP treatment for 24 h in resistant cells [SGC‐R (left) and BGC‐R (right)], CHOP mRNA level was detected by qRT‐PCR. (E) CHOP mRNA level was measured by qRT‐PCR in sensitive cells [SGC7901 (up) and BGC823 (down)] with ATF4 knockdown and DDP (1.2 μg/ml) incubation for 24 h. (F) Cell viability of SGC7901 (up) or BGC823 (down) cells with CHOP knockdown and DDP treatment for 24 h was measured by MTS assay

To understand how ATF4 regulates apoptosis under drug treatment, we compared the enriched ATF4 target genes (10 or 12 genes from the two ATF4 target gene signatures respectively) with the enriched apoptosis‐related genes (131 genes from the apoptosis related gene signature) (Figure S3A). Two candidates, CHOP (also known as DDIT3) and ATF6, were screened (Figure 3A). Further qRT‐PCR analysis confirmed that CHOP was the dominantly apoptosis‐related gene upregulated in sensitive cells upon DDP incubation, while ATF6 had little response (Figures 3B and S3B). And DDP could also induce the expression of CHOP downstream pro‐apoptotic genes such as BAD, BAX and BIM (Figure S3C). Meanwhile, ChIP analysis revealed that DDP significantly promoted the binding of ATF4 to the CHOP promoter region in sensitive cells, implying that ATF4 may activate CHOP transcription under DDP incubation (Figure 3C). CHOP protein expression was also downregulated in resistant cells (Figure S3D), and restoration of ATF4 expression in chemoresistant cells activated CHOP transcription and increased CHOP protein expression under DDP incubation (Figures 3D and S4D). In contrast, ATF4 knockdown in sensitive cells inhibited the upregulation of CHOP mRNA and protein level induced by DDP (Figures 3E and S3E), although the efficiency varied in different cells. What's more, knockdown of CHOP expression in sensitive cells dramatically attenuated DDP‐induced cell viability inhibition (Figure 3F), PARP1 cleavage (Figure S3F), and BAX and BIM mRNA expression (Figure S4A, B), indicating that knockdown of CHOP reduced DDP‐induced apoptosis. In addition, knockdown of CHOP expression in resistant cells reversed the effect of ATF4 over‐expression on viability inhibition and PARP1 cleavage induced by DDP treatment (Figure S4C, D). In summary, these results confirmed that ATF4 transcriptionally upregulates CHOP to stimulate drug‐induced apoptosis in gastric cancer cells.

### 2.4 β‐TrCP enhances ubiquitination‐dependent degradation of ATF4 in chemoresistant cells

**FIGURE 4:**
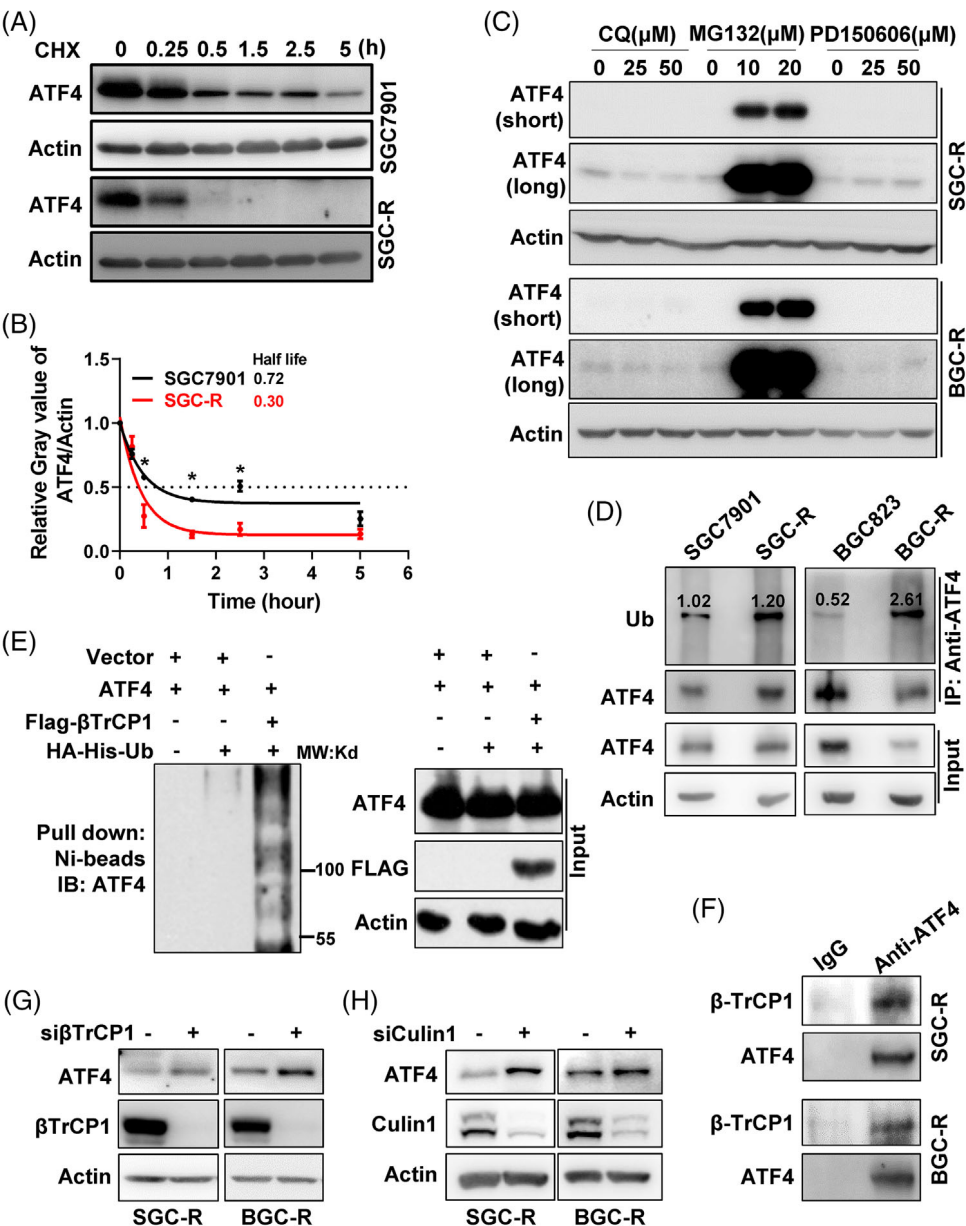
βTrCP‐enhanced ubiquitination‐dependent proteasomal degradation of ATF4 in chemoresistant cells. (A) Turnover of endogenous ATF4 in SGC7901 or SGC‐R cells under protein synthesis inhibitor cycloheximide (CHX, 50 μg/ml) incubation was detected by western blotting using anti‐ATF4 antibody. (B) The relative grey value of ATF4 compared to Actin in ‘A’ was analysed by Image J, which was further normalised to the ‘0’ time point sample. The fitted curves were drawn with GraphPad software and the half‐life of ATF4 turnover was analysed and shown. (C) Expression of ATF4 in SGC‐R (up) or BGC‐R (down) with different protein degradation inhibitors (lysosome inhibitor: CQ, proteasome inhibitor: MG132 and calpain inhibitor: PD150606) treatment was determined by western blotting. The short‐time and long‐time exposure results were shown as ATF4 (short) and ATF4 (long), respectively. (D) After pre‐treated with MG132, ubiquitination of endogenous ATF4 in sensitive or resistant cells was detected by anti‐ATF4 immunoprecipitation (IP) and anti‐Ub immunoblot. ATF4 ubiquitination was normalised to immunoprecipitated ATF4, and the normalised ratio was shown. (E) βTrCP1‐mediated ATF4 ubiquitination was determined by an in vitro ubiquitination assay in HEK293T cells. (F) The interaction of ATF4 with βTrCP1 in SGC‐R (up) or BGC‐R (down) cells pre‐treated with MG132 was analysed by anti‐ATF4 co‐immunoprecipitation (co‐IP), followed by anti‐βTrCP1 and anti‐ATF4 immunoblot. (G) Expression of ATF4 in SGC‐R (left) or BGC‐R (right) with βTrCP1 knockdown was determined by western blotting. (H) Expression of ATF4 in SGC‐R (left) or BGC‐R (right) with Cullin1 knockdown was detected by western blotting

Next, we aimed to determine how ATF4 is downregulated in chemoresistant cells. eIF2α phosphorylation has been reported to activate ATF4 translation under various conditions.^18^ However, eIF2α phosphorylation was not decreased in chemoresistant cells compared to sensitive cells (Figure S5A). Moreover, there was no significant difference in ATF4 mRNA levels between chemosensitive and chemoresistant cells (Figure S5B). Interestingly, the half‐life of endogenous ATF4 protein was shortened in chemoresistant cells compared to sensitive cells under protein synthesis inhibitor cycloheximide (CHX) treatment (Figure 4A, B). Meanwhile, turnover of exogenous ATF4 was also increased in resistant cells compared to sensitive cells (Figure S5C, D), indicating that ATF4 protein stability was decreased in chemoresistant cells. The proteasome degradation inhibitor MG132, rather than lysosome inhibitor CQ or calpain inhibitor PD150606, significantly restored the protein expression of ATF4 in chemoresistant cells (Figure 4C). Though it was reported that MG132 could induce ER stress to augment ATF4 expression,^27^ we further confirmed that MG132 retarded ATF4 protein turnover under CHX treatment (Figure S5E). These results suggested that the ubiquitin‐proteasome system is responsible for ATF4 degradation in chemoresistant cells. In fact, the ubiquitination level of endogenous ATF4 under MG132 treatment was indeed significantly increased in resistant cells compared to sensitive cells (Figure 4D).

Previous studies have shown that the E3 ubiquitin ligase SCF‐βTrCP complex directly interacts with ATF4 via the canonical βTrCP recognition motif DSGXXXS (218‐DSGICMS‐224 for ATF4) to catalyse ATF4 polyubiquitination and induce its ubiquitination‐dependent proteasomal degradation.^23^ As expected, βTrCP1 promoted ATF4 ubiquitination by in vitro ubiquitination assay (Figure 4E). In addition, ATF4 did interact with βTrCP1 in chemoresistant cells pre‐treated with MG132 (Figure 4F). Knockdown of SCF‐βTrCP complex, either βTrCP, Cullin1 or RBX1, significantly restored ATF4 protein expression in chemoresistant cells (Figures 4G, H and S5F). Together, these results indicated that SCF‐βTrCP E3 ligase is responsible for ATF4 ubiquitination and promotes its proteasomal degradation in chemoresistant cells.

### 2.5 CK1δ phosphorylates ATF4 to stimulate its ubiquitination‐dependent proteasomal degradation

**FIGURE 5:**
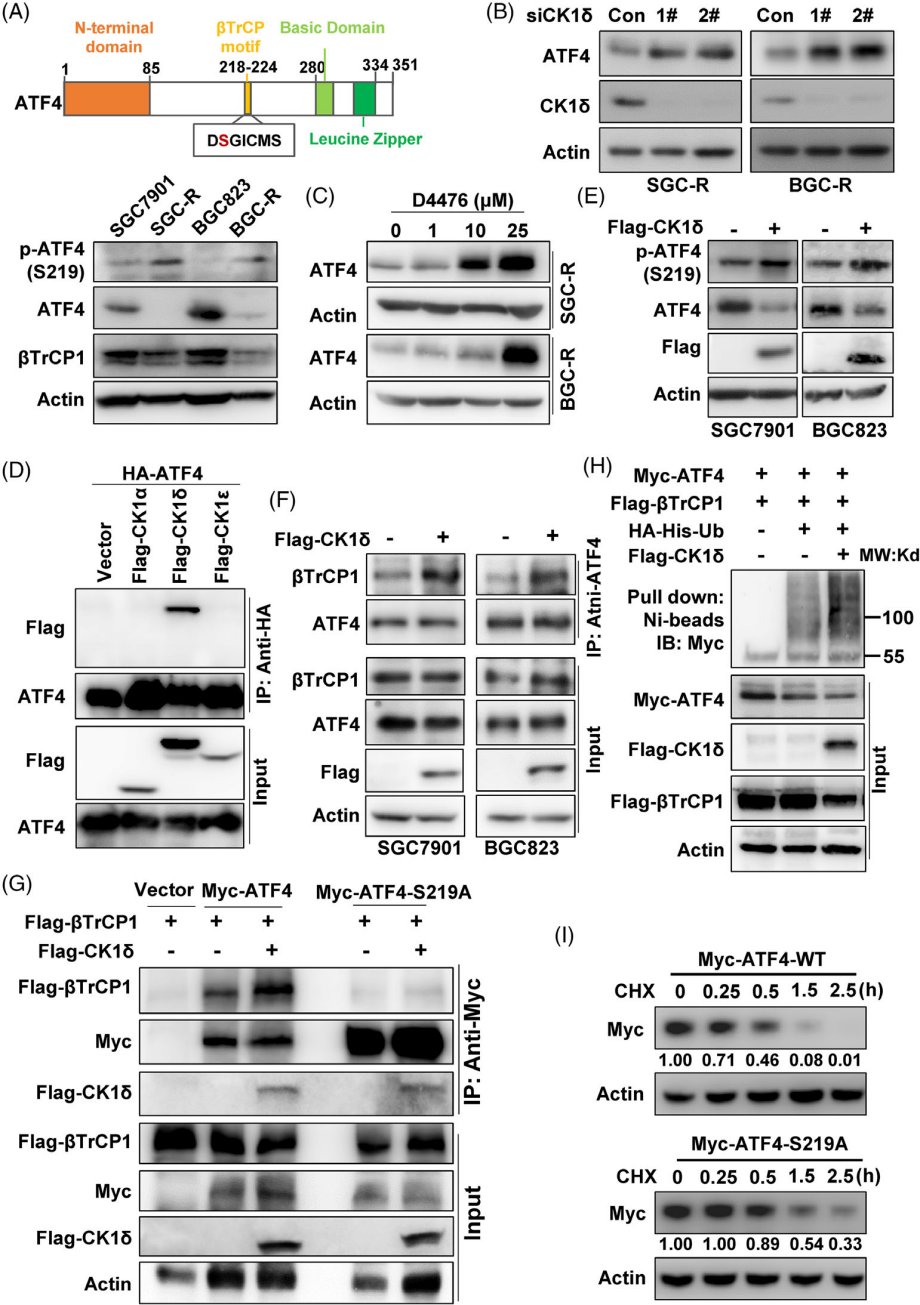
CK1δ phosphorylates ATF4 to stimulate its ubiquitination‐dependent proteasomal degradation. (A) The scheme of ATF4 protein was shown, and expression of phosphorylated ATF4‐S219 [p‐ATF4 (S219)], total ATF4 and βTrCP1 in sensitive and resistant cells were determined with western blotting. Expression of ATF4 in SGC‐R (left) or BGC‐R (right) with CK1δ knockdown (B) or inhibition (C) by D4476 was detected by western blotting. (D) Co‐IP was performed with anti‐HA antibody in HEK293T cells with HA‐ATF4 and Flag‐CK1α/δ/ε co‐transfection, and the interaction was detected by western blotting with anti‐Flag and anti‐ATF4 antibodies. (E) p‐ATF4(S219) in SGC7901 (left) or BGC823 (right) with Flag‐CK1δ over‐expression was analysed by western blotting. (F) After Flag‐CK1δ transfection, cells were pre‐treated with MG132, and interaction of ATF4 with βTrCP1 in SGC7901 (left) or BGC823 (right) cells was analysed by anti‐ATF4 co‐IP, followed by anti‐βTrCP1 and anti‐ATF4 immunoblot. (G) Interaction of wild‐type Myc‐ATF4 (Myc‐ATF4‐WT) or Myc‐ATF4‐S219A mutant with Flag‐βTrCP1 with/without Flag‐CK1δ co‐transfection in HEK293T cells was determined with anti‐Myc co‐IP, followed by anti‐Flag and anti‐Myc immunoblot. (H) βTrCP1‐mediated ATF4 ubiquitination in HEK293T cells with/without Flag‐CK1δ over‐expression was measured by in vitro ubiquitination assay. (I) Protein turnover of exogenous Myc‐ATF4‐WT or Myc‐ATF4‐S219A in SGC‐R cells under CHX (50 μg/ml) treatment with indicated times was detected by western blotting using anti‐Myc antibody. The relative grey value of ATF4 compared to Actin was analysed, and the normalised expression ratio was shown

Recent studies have shown that the phosphorylation of S219 in the βTrCP recognition motif is important for βTrCP‐mediated ATF4 polyubiquitination.^17^, ^23^ We confirmed that ATF4‐S219A (serine mutated to alanine), the phosphorylation deficient mutant of S219, lost its ability to interact with βTrCP1 (Figure S6A). As a result, βTrCP1‐mediated ubiquitination of ATF4‐S219A was dramatically decreased compared to wild type ATF4 (ATF4‐WT) (Figure S6B). Interestingly, S219 phosphorylation of ATF4 [p‐ATF4 (S219)], but not βTrCP1 expression, was upregulated in chemoresistant cells (Figure 5A), indicating that increased p‐ATF4 (S219) likely promotes βTrCP‐mediated ubiquitination and subsequent proteasomal degradation of ATF4 in chemoresistant cells. To further investigate the details of ATF4 S219 phosphorylation, GPS online software (http://gps.biocuckoo.cn/online.php) was applied to screen potential phospho‐kinases for ATF4 S219, which revealed CK1δ, cyclin‐dependent kinase 1 (CDK1), and CDK2 as potential candidates (Figure S6C). We found that knockdown of CK1δ rather than CDK1 or CDK2 significantly increased the protein expression of ATF4 in chemoresistant cells (Figures 5B and S6D), and knockdown of CK1δ could not upregulate ATF4 mRNA level (Figure S6E). Moreover, both D4476 (an inhibitor of CK1α and CK1δ) and IC261 (an inhibitor of CK1α, CK1δ and CK1ε), could restore ATF4 protein expression in resistant cells (Figures 5C and S6F). Furthermore, co‐immunoprecipitation (co‐IP) analysis showed that only CK1δ, but neither CK1α nor CK1ε, could interact with ATF4 (Figures 5D and S6G). Consistently, over‐expression of CK1δ in sensitive cells significantly increased p‐ATF4 (S219) and reduced ATF4 protein levels (Figure 5E), but CK1α or CK1ε over‐expression had little effect on ATF4 expression (Figure S6H). In addition, CK1δ over‐expression in sensitive cells increased the interaction of ATF4 with βTrCP1 with MG132 treatment (Figure 5F). Similarly, CK1δ over‐expression could potentiate the interaction of exogenous wild type ATF4 (ATF4‐WT) and βTrCP1, but this effect was not observed for ATF4‐S219A mutant (Figure 5G). Moreover, the in vitro ubiquitination assay confirmed that CK1δ promoted the polyubiquitination of ATF4 (Figure 5H). In line with the above findings, we further confirmed that over‐expressed CK1δ accelerated ATF4 protein turnover in SGC7901 cells under protein synthesis inhibitor CHX treatment (Figure S6I). And compared to ATF4‐WT, the protein turnover of ATF4‐S219A mutant was much slower in SGC‐R cells under CHX incubation (Figure 5I). These results suggested that CK1δ mediates ATF4‐S219 phosphorylation to enhance its interaction with βTrCP, thus promoting its ubiquitination and subsequent proteasomal degradation during chemoresistance in gastric cancer.

### 2.6 Stabilisation of ATF4 reverses chemoresistance

**FIGURE 6:**
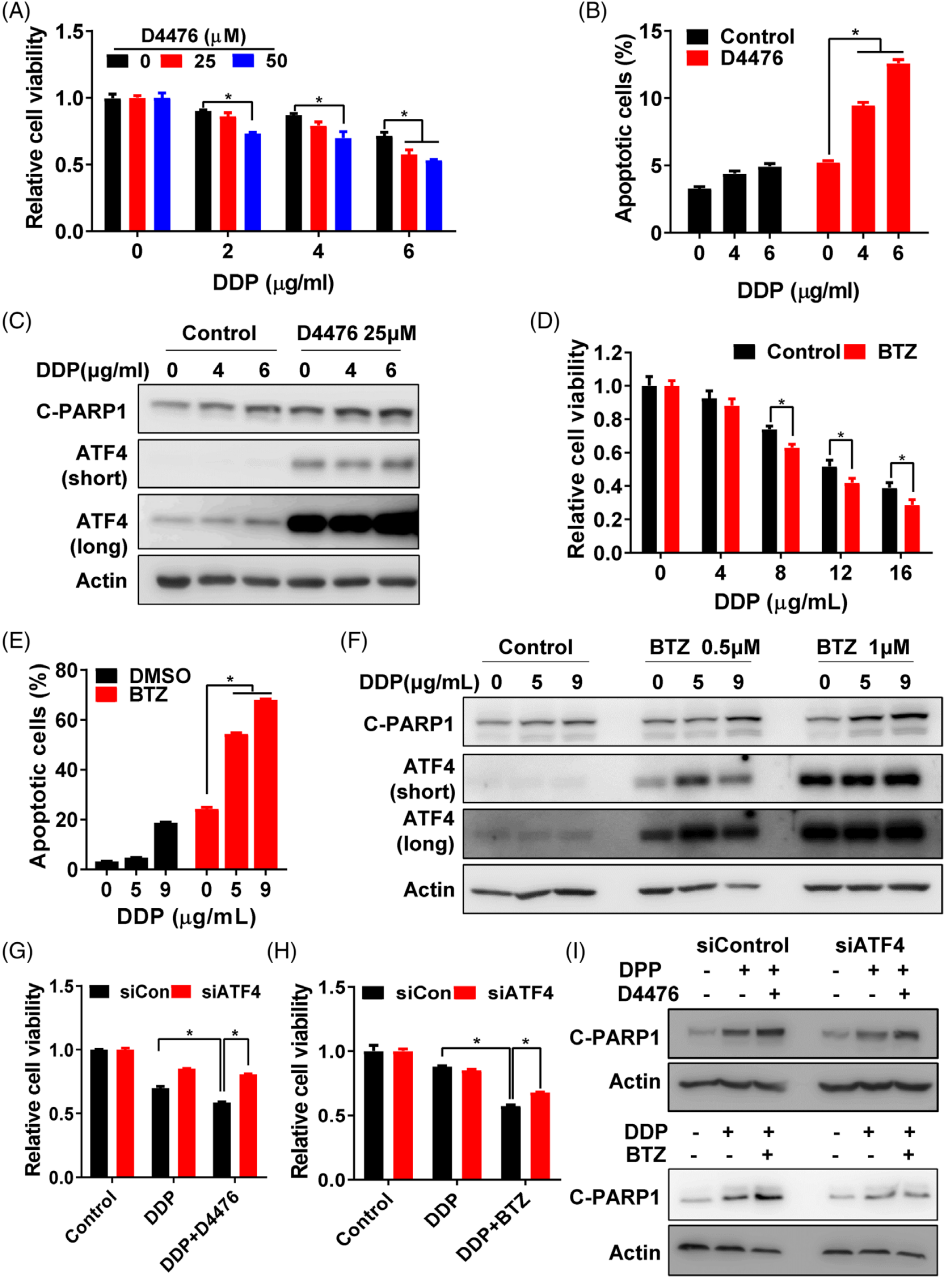
Stabilisation of ATF4 reverses chemoresistance. (A) Cell viability of SGC‐R cells with DDP treatment, with/without D4476 (0, 25 or 50 μM) combination for 24 h was measured by MTS assay. (B) Apoptosis of SGC‐R cells with DDP treatment, with/without D4476 (25 μM) combination for 24 h was analysed by PI/Annexin V double staining. (C) Expression of apoptosis marker C‐PARP1 and ATF4 in SGC‐R cells with DDP treatment, with/without D4476 combination for 24 h was detected by western blotting. The short‐time and long‐time exposure results were shown as ATF4 (short) and ATF4 (long), respectively. Cell viability (D) and apoptosis (E) of SGC‐R cells with DDP treatment, with/without BTZ (1 μM) combination for 24 h was measured. (F) Expression of apoptosis marker C‐PARP1 and ATF4 in SGC‐R cells with DDP treatment, with/without BTZ (0.5 or 1 μM) combination for 24 h was detected by western blotting. The short‐time and long‐time exposure results were shown as ATF4 (short) and ATF4 (long), respectively. After ATF4 knockdown, cell viability of SGC‐R cells with DDP (5 μg/ml) treatment, with/without D4476 (25 μM) (G) or BTZ (1μM) (H) combination for 24 h was measured by MTS assay. (I) After ATF4 knockdown, expression of C‐PARP1 in SGC‐R cells with DDP (5 μg/ml) treatment, with/without D4476 (up) or BTZ (down) combination for 24 h was detected by western blotting

The above data revealed that ubiquitination‐dependent proteasomal degradation of ATF4 played an important role in chemoresistance by inhibiting drug‐induced apoptosis. Therefore, we speculated that restoring ATF4 expression by inhibiting proteasomal degradation or CK1δ activity may be a potential strategy to reverse chemoresistance in gastric cancer. As CK1α had little effect on ATF4 expression (Figure S6H), the dual CK1α and CK1δ inhibitor D4476 was selected to explore the effect of ATF4 protein stabilisation on chemoresistance. As expected, D4476 could re‐sensitise chemoresistant cells to DDP by increasing viability inhibition (Figures 6A and S7A), elevating DDP‐induced apoptosis (Figures 6B, S7B and S8A) and enhancing DDP triggered PAPR1 cleavage (Figures 6C and S7C), illustrating that D4476 could reverse chemoresistance in gastric cancer cells. Meanwhile, bortezomib (BTZ), a proteasome inhibitor that has been approved by FDA for the clinical treatment of multiple myeloma (MM),^28^ was used to restore ATF4 expression in chemoresistant cells. Indeed, BTZ strengthened viability inhibition triggered by DDP (Figures 6D and S7D), increased DDP‐induced apoptosis (Figures 6E, S7E, and S8B), as well as restored ATF4 expression and augmented PARP1 cleavage (Figures 6F and S7F), indicating that BTZ could re‐sensitise DDP‐induced apoptosis in resistant cells. Though high dose of DDP (9 μg/ml) might induce apoptosis independent of ATF4, such as increasing DNA damage, BTZ significantly augmented DDP‐induced apoptosis (Figure 6E, F). Furthermore, knockdown of ATF4 expression in resistant cells obviously attenuated D4476 or BTZ reversed cell viability inhibition (Figures 6G, H and S7G, H), and reduced D4476 or BTZ‐enhanced PARP1 cleavage (Figures 6I and S7I) under DDP treatment. Overall, restoring ATF4 protein expression by CK1δ inhibitor or proteasomal degradation inhibitor reversed chemoresistance.

### 2.7 Dynamic ATF4 protein degradation in adaptive chemotherapy

**FIGURE 7:**
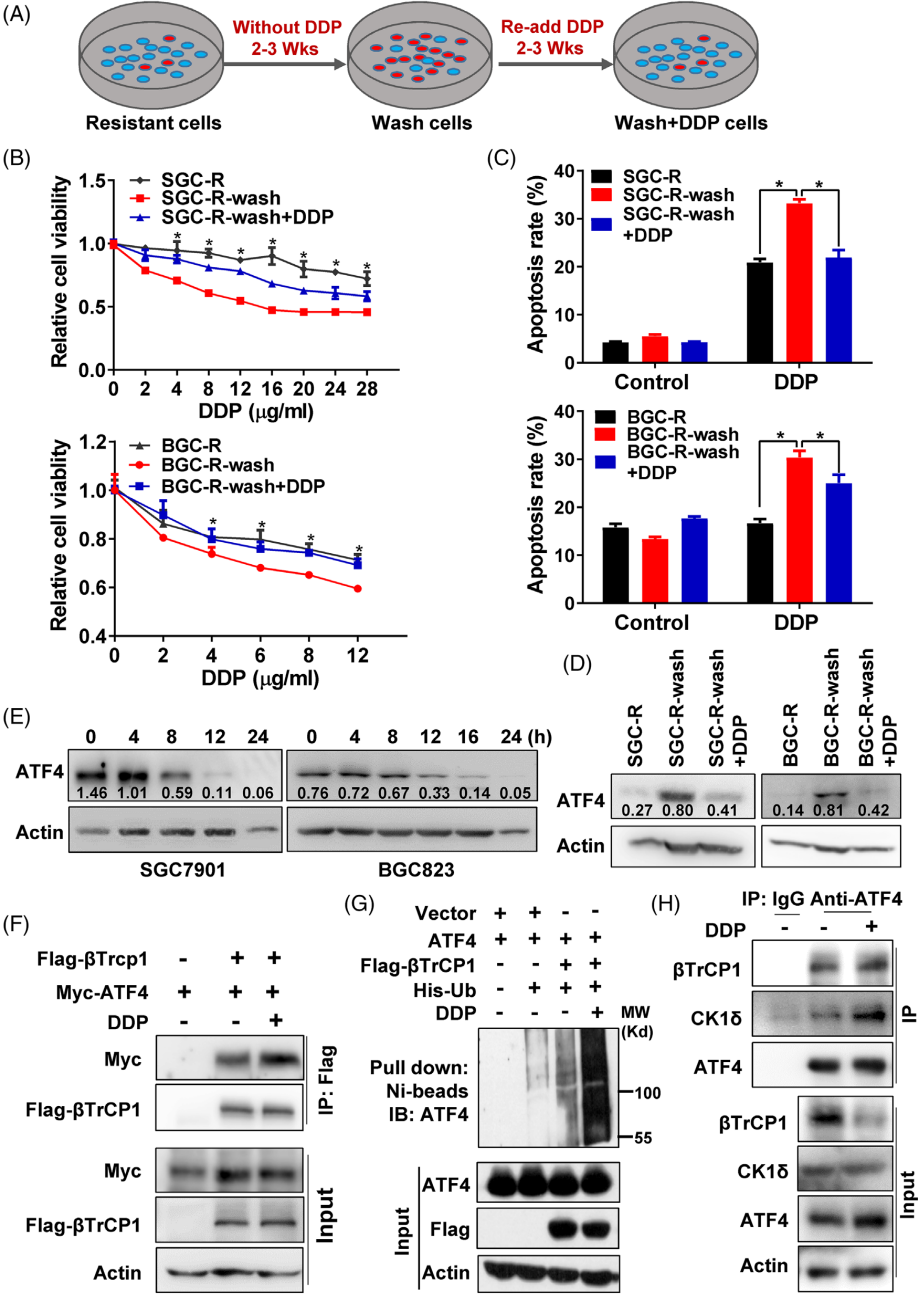
Dynamic ATF4 protein degradation in adaptive chemotherapy. (A) The process of drug withdraw and re‐addition for resistant cells maintaining was shown. Generally, the resistant cells were maintained in DDP free medium for 2–3 weeks to release the stress of DDP, which was named as SGC‐R wash and BGC‐R wash cells. Then DDP containing medium was used to re‐stress SGC‐R wash and BGC‐R wash cells for additional 2–3 weeks, and these cells were named as SGC‐R wash+DDP and BGC‐R wash+DDP. The sensitivity (B) and apoptosis (C) of different medium maintaining resistant cells as indicated to DDP for 24 h was measured. (D) Expression of ATF4 in resistant cells cultured with different medium as indicated was detected by western blotting. (E) Expression of ATF4 in sensitive cells (left for SGC7901 and right for BGC823) with time‐course DDP (1.2 μg/ml) treatment was determined by western blotting. (F) Interaction of Myc‐ATF4 with Flag‐βTrCP1 in HEK293T with/without DDP treatment for 12 h was determined by anti‐Flag co‐IP, and followed with anti‐Myc and anti‐Flag immunoblot. (G) The ubiquitination level of ATF4‐mediated by βTrCP1 with/without DDP treatment for 12 h was measured by in vitro ubiquitination assay. (H) Interaction of ATF4 with βTrCP1 and CK1δ in SGC7901 cells with/without DDP treatment for 12 h was determined by anti‐ATF4 co‐IP, then immunoblot with anti‐βTrCP1 and anti‐CK1δ

In addition to the acquisition of resistance‐conferring genetic mutations, increasing evidences have shown that chemoresistance could be resulted from non‐heritable mutations including epigenetic alterations such as DNA methylations, which could be reversible and dynamic upon drug withdraw or re‐adminstration.^6^ As shown in Figure 7A, resistant cells cultured with DDP free medium for 2–3 weeks, referred as SGC‐R‐wash or BGC‐R‐wash cells, restored drug sensitivity by showing increased viability inhibition and apoptosis activation after DDP treatment (Figures 7B, C and S9A). However, while re‐stressed SGC‐R‐wash or BGC‐R‐wash cells with DDP containing medium (defined as SGC‐R‐wash+DDP or BGC‐R‐wash+DDP cells) gradually acquired chemoresistance by attenuating viability inhibition and reducing apoptosis upon DDP treatment (Figures 7B, C and S9A). These results indicated that the chemoresistance in gastric cancer cells could be dynamic and reversible, depending on the presence of chemotherapeutic stress.

Interestingly, the expression of ATF4 protein level was negatively correlated to the reversible drug resistance (Figure 7D), indicating that chemoresistance in gastric cancer cells might be attributed to dynamic change of ATF4 protein expression. Indeed, depleting ATF4 expression in SGC‐R‐wash or BGC‐R‐wash cells remarkably decreased DDP‐induced viability inhibition (Figure S9B, C). Moreover, the protein level of ATF4 in sensitive cells decreased gradually in a time‐ and dose‐dependent manner under DDP incubation (Figures 7E and S9D), while the mRNA level of ATF4 did not decrease significantly (Figure S9E). Interestingly, DDP enhanced the interaction between exogenous βTrCP1 and ATF4, which eventually increased βTrCP1‐mediated ATF4 polyubiquitination (Figure 7F, G). And inhibition of CK1δ by D4476 impaired DDP‐induced interaction of βTrCP1 with ATF4 (Figure S9F). In addition, DDP promoted the interaction of ATF4 with βTrCP1 and CK1δ in sensitive cells with MG132 pre‐treatment (Figures 7H and S9G). Furthermore, both ATF4 polyubiquitination and p‐ATF4 (S219) were elevated in sensitive cells treated with DDP (Figure S9H, I). Taken together, these findings suggested that ATF4 turnover, which is regulated by CK1δ and βTrCP, is important for dynamic chemoresistance in gastric cancer.

### 2.8 ATF4 stabilisation by BTZ or CK1δ inhibitor enhances the efficacy of chemotherapy in gastric cancer

**FIGURE 8:**
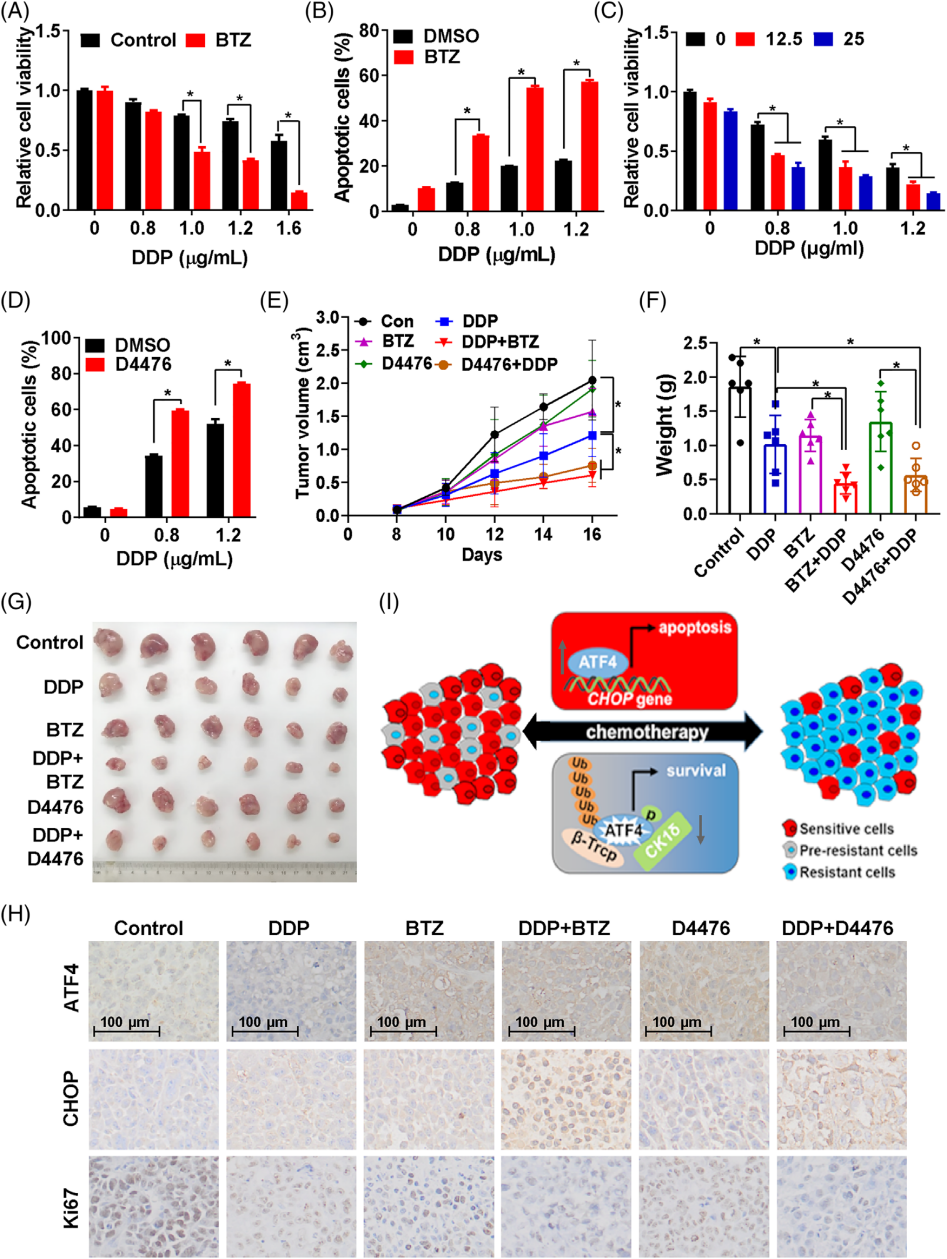
ATF4 stabilisation by BTZ or CK1δ inhibitor enhances the efficacy of chemotherapy in gastric cancer. Viability (A) and apoptosis (B) of SGC7901 cells treated by DDP with/without BTZ (0.1μM) for 24 h was measured. (C) Viability of SGC7901 cells treated by DDP and D4476 (0, 12.5 or 25 μM) for 24 h was measured by MTS assay. (D) Apoptosis of BGC823 cells treated by DDP and D4476 (25μM) for 24 h was measured. Xenograft model was performed with SGC7901 cells under indicated treatments, tumour growth curve (E) was plotted according to the tumour volume measured every other day, tumour weight (F) and tumour photos (G) were obtained at the end of the experiment. (H) Expression of ATF4, CHOP and Ki67 in different groups as indicated were analysed by IHC. (I) Proposed model. Generally, chemotherapeutics could promote ATF4 to activate CHOP transcription, thus inducing cell apoptosis. Simultaneously, chemotherapeutics increases ATF4‐S219 phosphorylation via CK1δ, which subsequently enhances βTrCP‐mediated ATF4 poly‐ubiquitination‐dependent proteasomal degradation, eventually resulting in chemoresistance. Therefore, this acquired resistance is a dynamic reversible process. Upon drug withdraw, ATF4 expression gradually recovers and resistant cells will re‐sensitise to chemotherapeutics

Therefore, we hypothesised that ATF4 stabilisation by BTZ or CK1δ inhibitor could synergise with DDP to enhance its toxicity to gastric cancer. Indeed, BTZ significantly increased DDP‐induced viability inhibition, apoptosis activation and PAPR1 cleavage by stabilising ATF4 protein expression (Figures 8A, B, S10A–D and S11A). Additionally, knockdown of ATF4 expression greatly impaired BTZ augmented DDP‐induced PARP1 cleavage (Figure S10E, F), indicating that BTZ enhanced the toxicity of DDP in gastric cancer cells depending on ATF4‐activating apoptosis. Similar to BTZ, the CK1δ inhibitor D4476 was able to increase cytotoxicity of DDP in sensitive cells by increasing DDP‐induced viability inhibition and apoptosis activation (Figures 8C, D, S10G and S11B). In the nude mice xenograft model using SGC7901 cells, combination of BTZ or D4476 with DDP retarded tumour growth more effectively compared to DDP only group (Figure 8E–G), while the body weights of mice in each group were not significantly different at the end of the experiment (Figure S10H). Moreover, expression of ATF4 was reduced in DDP only group, while BTZ or D4476 stabilised ATF4 protein expression in DDP combination groups (Figure 8H). In line with ATF4 expression, CHOP protein expression was increased in BTZ or D4476 and DDP combination group (Figure 8H). Besides, Ki67 staining indicated that in vivo cell proliferation was suppressed more profoundly in combination groups (Figure 8H). These data supported that the combination of DDP with CK1δ inhibitor or BTZ may be a new strategy to sensitise chemotherapy in gastric cancer.

## 3 DISCUSSION

Chemoresistance remains a major obstacle to successful cancer therapy. The standard strategy for chemoresistance is to change the therapeutic regimen because chemoresistance is usually recognised as a stable and heritable state. However, over the past decades, increasing evidences have shown that epigenetic alterations are also involved in chemoresistance, which could be unstable and reversible.^1^, ^6^ In the present study, we found that ATF4 ubiquitination‐dependent proteasomal degradation regulated by CK1δ and βTrCP is important for dynamic and reversible chemoresistance in gastric cancer. ATF4 downregulation attenuated drug‐induced apoptosis by suppressing CHOP transcription. ATF4 stabilisation by proteasome or CK1δ inhibitor overcame chemoresistance and improved the efficacy of chemotherapy both in vitro and in vivo (Figure 8I). Previous studies had found several other mechanisms implicated in cisplatin resistance. Thus, downregulation of ATF4 at least partially contributes to cisplatin resistance in gastric cancer cells. More importantly, the dynamic ATF4 turn over determined dynamic chemoresistance.

Cisplatin containing regimen is still the commonly used chemotherapeutic in gastric cancer. It is well documented that multiple mechanisms are implicated in cisplatin resistance, including upregulated p‐glycoprotein expression,^12^ enhanced DNA damage response,^13^, ^14^ and reprogrammed energy metabolism.^15^ Nevertheless, there are limited effective approaches to overcome cisplatin resistance in clinic to date. Other reports have shown that ATF4 mRNA is upregulated in several cancer cells, including lung and prostate cancer cells, under cisplatin treatment, transactivating the antioxidant response and autophagy pathways to promote chemoresistance.^29^, ^30^ However, we confirmed that ATF4 is downregulated in cisplatin‐resistant gastric cancer cells. ATF4 over‐expression in resistant cells promoted while ATF4 depletion in sensitive cells attenuated drug‐induced apoptosis by regulating CHOP transcription (Figures 1, 2, 3). Meanwhile, the alteration of ATF4 in gastric cancer cells had little effect on the expression of antioxidant and autophagy related genes before or after DDP incubation (data not shown). However, transient incubation with low dose (0.1–0.4 μg/ml) cisplatin increased ATF4 expression (data not shown), while transient incubation with a relatively high dose (>0.8 μg/ml), gradually reduced ATF4 expression in gastric cancer cells (Figure 7). Therefore, it seems that the role and mechanism of ATF4 in cisplatin resistance could be complicated and context dependent. In particular, ATF4 expression is elevated in some tumours, such as breast cancer and colorectal cancer, compared to matched normal tissues, especially in hypoxic and nutrient deficient regions of tumour tissues.^31^, ^32^ And depletion of ATF4 significantly inhibits colorectal cancer cell growth in vitro and in vivo.^32^ Therefore, ATF4 may function to promote metabolic homeostasis and enable cancer cell survival under certain stress conditions. Interestingly, the established resistant cells usually grew much slower than the sensitive parental cells, indicating that chemoresistance is an adaptive strategy for cancer cells to survive at the price of rapid proliferation. Thus, more precise investigations and personalised strategies should be applied to overcome cisplatin resistance in various cancers.

ATF4 protein expression is regulated by various mechanisms in response to different stresses. For example, PKR‐like endoplasmic reticulum kinase (PERK) is activated during hypoxia, which induces eIF2α phosphorylation, followed by increased ATF4 protein translation to transactivate genes that restore ER function and enable cell survival.^33^, ^34^ Upon serine depletion, eIF2α phosphorylation is elevated by general control of amino acid biosynthesis protein 2 (GCN2) to enhance ATF4 protein synthesis, thus activating the transcription of genes required for serine synthesis.^35^, ^36^ On the other hand, ATF4 protein is selectively degraded in an ATF4‐S219 phosphorylation‐dependent manner via SCF‐βTrCP protein complex, a Skp1/Cullin1/F‐box family E3 ubiquitin ligase.^37^ Herein, we found that ubiquitination‐dependent proteasomal degradation of ATF4 was enhanced in chemoresistant cells (Figure 4). CK1δ was identified as the kinase responsible for ATF4‐S219 phosphorylation, and inhibition of CK1δ restored ATF4 expression to reverse chemoresistance in resistant cells. Moreover, the over‐expression of CK1δ in sensitive cells increased ATF4‐S219 phosphorylation and ATF4 ubiquitination, which ultimately reduced ATF4 expression (Figure 5). Our finding was consistent with previous report that CK1‐mediated mouse ATF4 S218 phosphorylation in the βTrCP degron, which is S219 in human, and promoted ATF4 ubiquitination‐dependent degradation during cell cycle progression and neurogenesis.^37^ However, how CK1δ‐dependent ATF4 phosphorylation is activated to promote chemoresistance remains unknown. Previous reports have revealed that DNA damage reagents could stimulate CK1δ phosphorylation via the activation of ataxia telangiectasia mutated (ATM).^38^, ^39^ We confirmed that the expression of CK1δ showed no significant difference in sensitive and resistant cells (Figure S11C). However, cisplatin treatment increased CK1δ phosphorylation (Figure S11D). Thus, cisplatin may also activate CK1δ to stimulate ATF4‐S219 phosphorylation by inducing DNA damage.

We speculated that chemotherapeutic drugs could simultaneously activate the prosurvival program in some cancer cells while inducing apoptosis or cell cycle arrest in other cancer cells. Therefore, we believe that resistant cancer cells do not pre‐exist but are somehow induced by treatment, which was similar to the scenario in bacteria, as they can transiently increase their toleration in the presence of antibiotics.^40^, ^41^ Interestingly, this adaptive mutability also takes place in human cancer cells. EGFR/BRAF inhibition can also promote drug resistance by downregulating mismatch repair (MMR) and homologous recombination (HR) DNA repair genes to induce genome instability and generate more mutations with adaptive advantages.^42^ Therefore, adding other drugs targeting this adaptive pathway might be rational for the design of new combination strategies in the future.

Clinically, re‐administration of the same chemotherapeutics, including platinum agents and EGFR TKIs, has shown promising effects after a ‘drug holiday’ with tumour progression.^1^, ^9^ Therefore, adaptive therapy has been proposed for effective cancer treatment,^43^ though the underlying mechanisms remain largely unknown. In this study, we found drug resistance to be a dynamic and reversible process resulting from dysregulated protein turnover rather than the acquisition of resistance‐conferring genetic mutations. We found that cisplatin withdrawal gradually re‐sensitised resistant cells to cisplatin (SGC‐R wash and BGC‐R wash cells), accompanied by the recovery of ATF4 protein expression. Depleting ATF4 expression retrieved drug resistance, even after cisplatin withdrawal (Figure 7), highlighting the dependence of drug resistance on low expression of ATF4. Therefore, we hypothesised that upon cisplatin treatment, activated CK1δ can stimulate ATF4‐S219 phosphorylation, which promotes ATF4 ubiquitination and subsequent proteasomal degradation. And cisplatin withdrawal inactivates CK1δ, resulting in decreased S219 phosphorylation and subsequent accumulation of ATF4 protein. In line with these findings, we further provided evidence that restoration of ATF4 expression by CK1δ activation inhibition or proteasomal degradation inhibitor successfully enhances cisplatin‐induced toxicity in gastric cancer.

In conclusion, CK1δ stimulates βTrCP‐dependent ATF4 polyubiquitination and subsequent proteasomal degradation to promote chemoresistance in gastric cancer. Stabilisation of ATF4 protein with proteasomal degradation inhibitor BTZ, which has been applied clinically for the treatment of multiple myeloma and other cancers, might be a rational strategy to improve chemotherapeutic efficacy in gastric cancer.

## 4 MATERIALS AND METHODS

### 4.1 Cell lines, antibodies and reagents

Gastric cancer cell lines SGC7901, BGC823, AGS and HGC27 were purchased from the Type Culture Collection of the Chinese Academy of Sciences (Shanghai, China). Drug‐resistant cells SGC‐R and BGC‐R cells were developed from SGC7901 and BGC823, respectively.^25^ The cells were cultured in RPMI 1640 (Invitrogen, 11875‐093, Shanghai, China) medium supplemented with 10% of foetal bovine serum (FBS), 100 U/ml of penicillin and 100 μg/ml of streptomycin (Life Technologies/Gibco, Shanghai, China), and 1 or 0.5 μg/ml of DDP was added in the culture medium for SGC‐R or BGC‐R cells maintaining. HEK293T cell was cultured in DMEM medium (Invitrogen, 12100‐046). The cells were grown at 37°C in a humidified incubator with 5% CO2 and 95% humidity.

The following antibodies were used for western blotting, or immuno‐precipitation (IP): ATF4 (rabbit, 11815), β‐TrCP (rabbit, 4394), β‐actin (rabbit, 4970), CDK1 (rabbit, 9111), CDK2 (rabbit, 18048), p‐eIF2α (rabbit, 3398), Myc (mouse, 2276) and cleaved PARP1 (C‐PARP1) (rabbit, 9541) were purchased from Cell Signaling Technology (Shanghai, China). p‐ATF4(S219) (rabbit, csb‐pa020025) was from CUSABIO (Wuhan, China). CHOP (Rabbit, 15204‐1‐AP) was obtained from Proteintech (Wuhan, China). CK1δ (mouse, sc‐55553) and Ub (Ubiquitin) (mouse, sc‐8017) were from Santa Cruz (Shanghai, China). Flag (mouse, F1804‐1) and HA (mouse, 11867423001) were from Merck/Sigma Aldrich (Shanghai, China). MG132 (474790) was purchased from Merck/Calbiochem. Bortezomib (BTZ) (S1013) and D4476 (S7642) were purchased from Selleck (Shanghai, China). Chloroquine (CQ) (C6628), PD150606 (D5946) and Cycloheximide (R750107) were purchased from Merck/Sigma Aldrich. Cisplatin (DDP) (HY‐17394) was purchased from MedChemExpress (Shanghai, China).

### 4.2 SiRNA and plasmid transfections

For siRNA transfections, cells were seeded overnight and transfected with Lipofectamine™ RNAiMAX transfection reagent (Thermo Fisher Scientific, 13778, USA) according to the manufacturer's protocol. For plasmid transfections, cells were seeded overnight, and plasmids were transfected with X‐treme GENE HP DNA Transfection Reagent (Merck/Sigma Aldrich, 06366236) according to the manufacturer's instructions. After 1–3 days, transfected cells were treated as indicated. The siRNA duplexes were purchased from Genepharma (Shanghai, China) and listed in Table S1. The plasmid expressing full‐length wild‐type HA‐ATF4 in pcDNA3.1‐HA vector was purchased from Genechem (Shanghai, China). Plasmids expressing Myc‐ATF4 or Flag‐ATF4 was constructed into pCMV3Tag vector. ATF4 S219A (Serine to Alanine) mutant was generated by Quick Change Site‐Directed Mutagenesis Kit (Agilent, 600674‐51, USA). Flag‐βTrCP1 and Flag‐CK1α/δ/ε were cloned into pCMV2‐Flag vector. And His‐Ub was cloned into pcDNA3.0‐His vector. Primers used were listed in Table S1.

### 4.3 RNA extract, reverse transcription and qPCR

Total RNA was extracted with TRIzol reagent (Invitrogen, 15596026) according to the manufacturer's instructions, and RNA concentration was quantified by NanoDrop 2000 (Nanodrop, Wilmington, DE, USA). RNA (1–2 μg) was reverse transcribed with the High Capacity cDNA Reverse Transcription Kit (Thermo Fisher Scientific, 4368813). The real‐time PCR (qPCR) was conducted by using SYBR Green Master Mix Kit (ComWin Biotech, CW0659s, Beijing, China). The qPCR data were normalised to the control group, and relative expression of indicated genes shown in the histograms were expressed as mean ± SD. The primers used in this study were listed in Table S1.

### 4.4 Western blotting

Generally, cell lysates were collected with 1× loading buffer containing 2% SDS, 0.1% bromophenol blue, 10% glycerinum, 1.5% DTT (dithiothreitol) and 0.1 M Tris‐HCl (pH 6.8). The cells lysates were boiled and separated by SDS‐PAGE, transferred to polyvinylidene fluoride (PVDF) membranes (Bio‐Rad, Shanghai, China), and then blocked with 5% non‐fat milk (dissolved with TBST). The membranes were incubated with the indicated primary antibodies at 4°C overnight and then incubated with the suitable secondary antibody conjugated with horseradish peroxidase (HRP) (111‐035‐003, Jackson Immuno Research, USA) at 37 °C for 2 h. Ultimately, the membranes were visualised in an Amersham Imager 600 system (GE Healthcare Life Science, Shanghai, China) with a Chemiluminescence Detection Kit for HRP (Biological Industries, Cromwell, USA, 20‐500‐120; FDbio, china, FD8030) to determine protein expression.

### 4.5 Cell growth assay and apoptosis detection

Around 7000–10 000 cells seeded overnight in a 96‐well plate were treated as indicated. The cell viability was measured by the CellTiter 96^®^ AQueous MTS Reagent (Promega, G1111A, USA). The absorbance of each well was detected by a microplate reader at 490 nm (OD490). OD490 was normalised to the control group, which referred as relative cell viability. And the relative cell viability was expressed as mean ± SD in the histograms. Cell apoptosis was measured by flow cytometry and western blotting. The FITC‐annexin V and propidium iodide (PI) double staining apoptosis kit (BD Biosciences, 556547, USA) was applied for flow cytometer analysis, cells treated as indicated for 24–72 h were harvested by trypsin and re‐suspended in 100 μl 1 × binding buffer, 5 μl of each staining reagent was then added to the cell suspension and incubated for 15 min at room temperature. The samples were analysed by BD FACSCalibur™ flow cytometer (BD Biosciences). The percentage of apoptotic cells in each group was shown as mean ± SD in the histograms.

### 4.6 Immunoprecipitation/Co‐immunoprecipitation

The cells were harvested and lysed in ice‐cold lysis buffer [20 mM Tris (pH 7.4), 150 mM NaCl, 0.5–1% (v/v) Triton™ X‐100] containing protease and phosphatase inhibitors (EDTA‐free Protease Inhibitor Cocktail, B14001, Selleck; Phosphatase Inhibitor Cocktail, B15002, Selleck). The cell extracts were centrifuged at 14 000 rpm at 4°C for 30 min, and the supernatant was incubated with indicated antibody or control IgG overnight at 4°C with rotation, then protein G agarose beads (17061802, GE Healthcare Life Science) were added and incubated for another 4 h. After washing, the precipitated samples were re‐suspended with 1× SDS loading buffer, boiled and sent for western blotting.

### 4.7 Chromatin immunoprecipitation (ChIP)

Cells seeded overnight in 10 cm dishes were treated as indicated. ChIP analysis was conducted with the SimpleChIP™ Enzymatic Chromatin IP Kit (Cell Signaling Technology, 91820s) as previously described.^44^ Antibodies used were anti‐ATF4 or control rabbit IgG. The primers used for the real‐time qPCR of precipitated DNA were shown in Table S1. And the ChIP qPCR data were normalised to IgG control group, and relative ATF4 enrichment was shown as mean ± SD in the histograms.

### 4.8 In vitro ubiquitination assay

HEK293T cells were transfected with constructs encoding Flag‐βTrCP1, His‐Ub and Myc‐ATF4 or Myc‐ATF4S219A mutant. After 48 h, cells were treated with MG132 (20 μM) for 6 h and then lysed with denatured buffer containing 6 M guanidine as described previously.^45^ ATF4‐ploy‐Ub was purified by Ni^+^‐beads (Qiagen, 30210, Germany) pull down and detected by immunoblot using Myc antibody. And the expression Flag‐βTrCP1 and Myc‐ATF4 or Myc‐ATF4‐S219A in transfected cells was detected by immunoblot.

### 4.9 Colony growth in Matrigel

Cells were seeded in 12‐well plates containing 200 μl of Matrigel (Corning BioCoat, 354234, USA) and 200 μl of medium with 10% of FBS (1:1). After 4 h, 1 ml complete medium was added gently to the layer of Matrigel. Cisplatin was added at second day, and cells were further maintained for 7 days. Cell growth was evaluated using an inverted microscope and colonies (diameter > 1 μm) were quantified using Image J software. Each assay was repeated at least three times. And the colony numbers were counted and expressed as mean ± SD, which was shown in the histograms.

### 4.10 Xenograft tumour growth in mice

SGC7901 cells but not GC823 cells was used for in vivo study since only SGC7901 cells could form tumours in nude mice. A total of 5 × 10^6^ SGC7901 cells re‐suspended in 0.1 ml 1× PBS were subcutaneously injected into flanks of 6‐week‐old BALB/c athymic nude mice (SPF Biotechnology, Beijing, China). Once the tumour volume reached about 100 mm^3^, mice were randomly divided into six groups (6 mice per group) and treated as follows: (1) DMSO as control group; (2) DDP (1 mg/kg) only, (3) BTZ (1 mg/kg) only, (4) DDP + BTZ, (5) D4476 (25 mg/kg) only, (6) DDP + D4476. All drugs were intraperitoneally injected every other day. And the tumour volume was measured on the same day before drug injection with vernier calliper as previous reported.^46^ Ten days after injection, mice were euthanised, tumours were harvested and weighed. Tumour volume at each time point was calculated with the following equation: *V* = (*Length* × *Width* ^2^)/2, tumour volume for each group was expressed as mean ± SD and the tumour curve was drawn accordingly. And ATF4, CHOP and Ki67 expression in tumour tissues were analysed by IHC.

### 4.11 Statistical analysis

Unless specifically stated, Student's *t*‐test was performed for statistical significance analysis. ‘*’ represents as *p* value < 0.05, which was considered as statistically significant, ‘#’ represents as *p* value > 0.05, and ‘N/A’ means not analysed.

## CONFLICT OF INTEREST

All authors declare no conflict of interest.

## Supporting information

Supporting Information
