# Supplementary material for: CK1δ stimulates ubiquitination‐dependent proteasomal degradation of ATF4 to promote chemoresistance in gastric Cancer
Source: Clin Transl Med. 2021 Oct 14;11(10):e587. doi: 10.1002/ctm2.587 (PMC8516343; doi:10.1002/ctm2.587)
Supplement: Supplementary file 1 — Supporting Information [file CTM2-11-e587-s001.docx]

**Supplemental Figures and Table**

Lifeng Feng^1,#^, Muchun Li^1,#^, Xinyang Hu^1^, Yiling Li^1^, Liyuan Zhu^1^, Miaoqin Chen^1^, Qi Wei^1^, Wenxia Xu^2^, Qiyin Zhou^1^, Weikai Wang^1^, Dingwei Chen^4^, Xian Wang^3,*^, Hongchuan Jin^1,*^

^1^Laboratory of Cancer Biology, Key lab of Biotherapy in Zhejiang, Cancer Center of Zhejiang University, Sir Run Run Shaw Hospital, Medical School of Zhejiang University, Hangzhou, China; ^2^Central laboratory, Affiliated Jinhua Hospital, Medical School of Zhejiang University, Jinhua, Zhejiang, China; ^3^Department of Medical Oncology, Sir Run Run Shaw Hospital, Medical School of Zhejiang University, Hangzhou, China; ^4^Department of General Surgery, Sir Run Run Shaw Hospital, Medical School of Zhejiang University, Hangzhou, China.

#: These two authors contribute to this work equally.

***Correspondence to**: Dr. Hongchuan Jin, jinhc@zju.edu.cn; Dr. Xian Wang, wangx118@zju.edu.cn


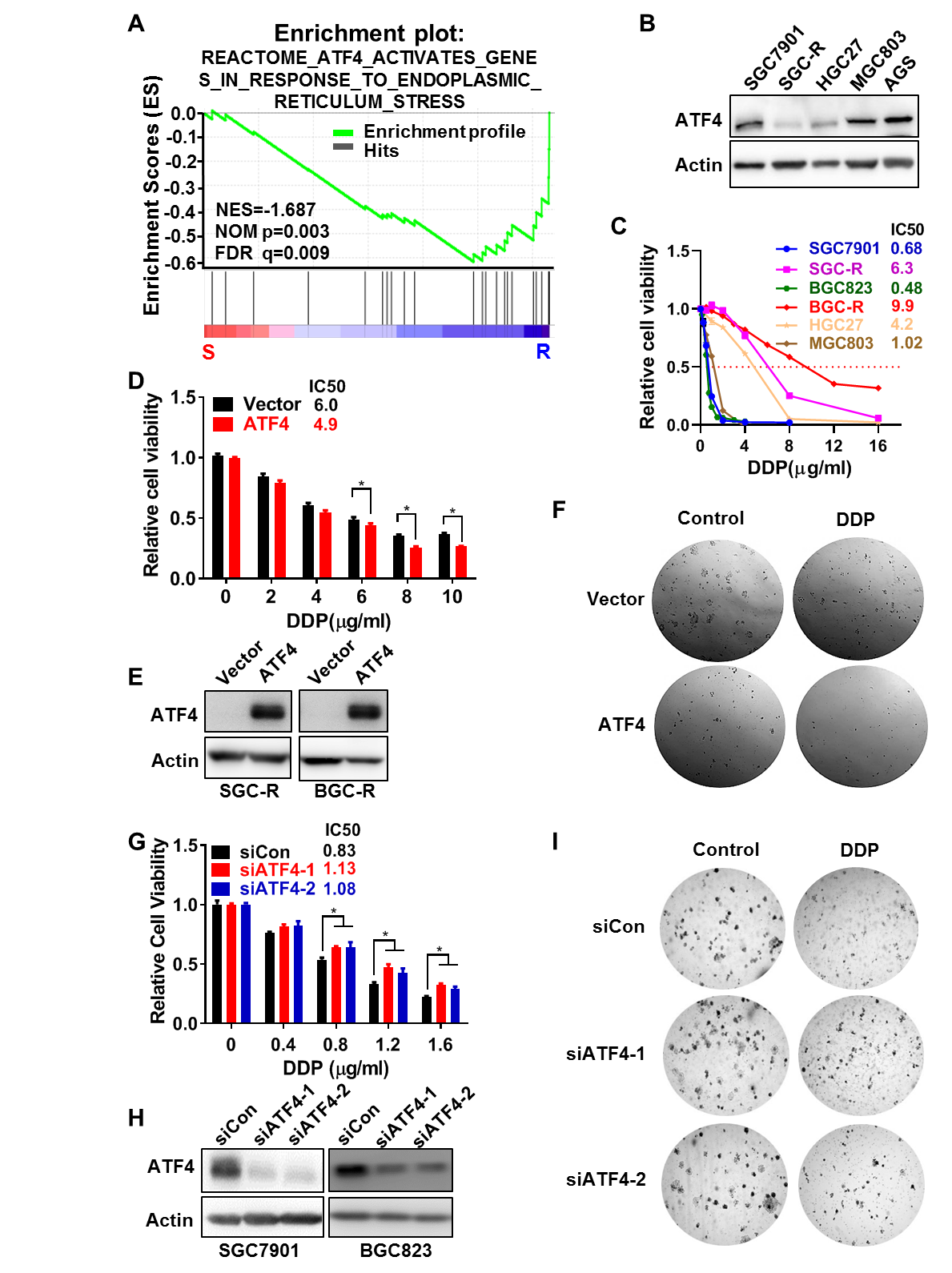


**Supplemental Figure 1. ATF4 is downregulated to promote chemoresistance in gastric cancer**

A. The gene expression profile was analyzed by Gene Set Enrichment Analysis (GSEA) in chemoresistant cells (SGC-R and BGC-R) and their parental sensitive cells (SGC7901 and BGC823), assessing the correlation to ATF4 target gene signature. B. The expression of ATF4 in gastric cancer cell lines was detected by western blot. C. Cell viability of SGC7901, SGC-R, BGC823, BGC-R, MGC803 and HCG27 under DDP treatment for 24 hours was measured by MTS assay. D. Viability of BGC-R cells with ATF4 over-expression and DDP treatment for 24 hours was measured by MTS assay. E. The over-expressed ATF4 in SGC-R or BGC-R cells was confirmed by western blotting. F. The represent photos of clonal growth of SGC-R cells with ATF4 over-expression, and DDP treatment for 7 days were shown. G. Cell viability of BGC823 cells with ATF4 knockdown and DDP treatment for 24 hours was measured by MTS assay. H. Knockdown of ATF4 in SGC7901 or BGC23 cells was validated by western blotting. I. The represent photos of clonal growth of SGC7901 cells with ATF4 knockdown, and DDP treatment for 7 days were shown.


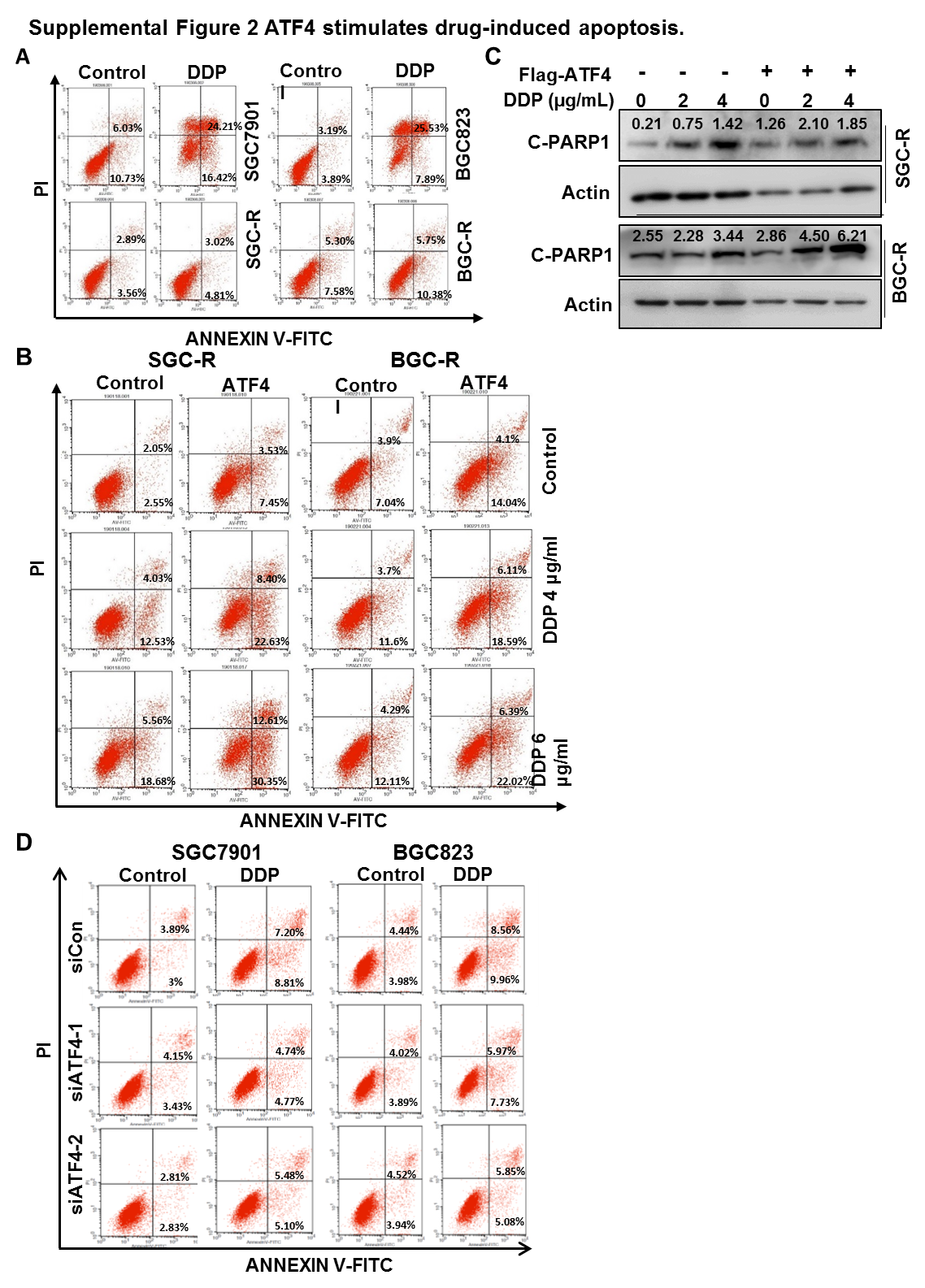


**Supplemental Figure 2. ATF4 stimulates drug-induced apoptosis**

A. The represent apoptosis pictures of SGC7901 and SGC-R or BGC823 and BGC-R under DDP (1.2μg/ml) incubation for 24 hours were shown. B. The represent apoptosis pictures of SGC-R or BGC-R cells with ATF4 overexpression and DDP (4μg/ml) treatment for 24 hours were shown. c. The expression of apoptosis marker cleave-PARP1 (C-PARP1) in SGC-R (up) or BGC-R (down) cells with ATF4 overexpression and DDP treatment for 24 hours was detected by western blotting. D. The represent apoptosis pictures of SGC7901 or BGC823 cells with siATF4s and DDP (1.2μg/ml) treatment for 24 hours were shown.


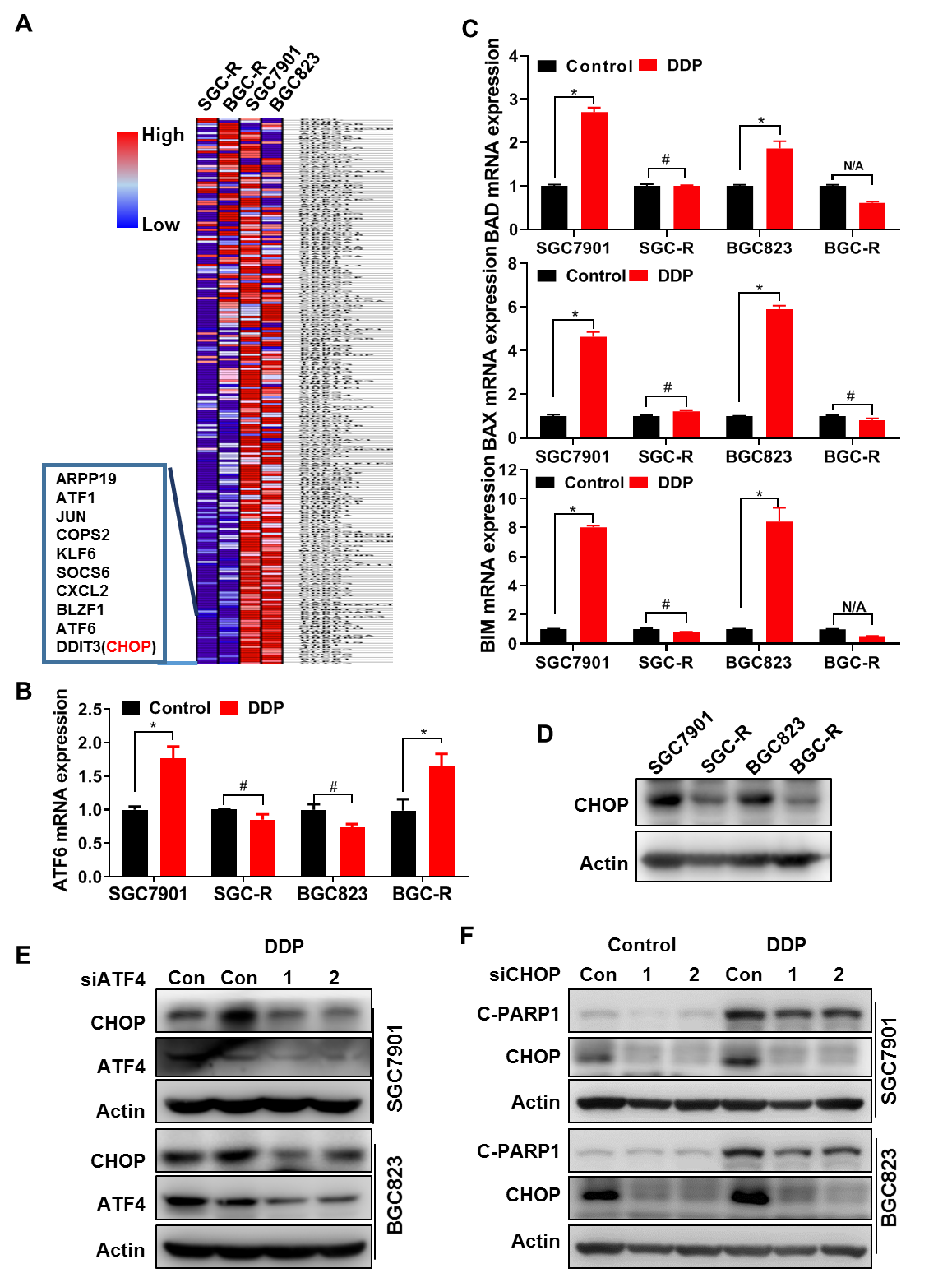


**Supplemental Figure 3. ATF4 stimulates drug-induced apoptosis by activating CHOP transcription**

A. Heatmap of apoptosis related genes enriched by GSEA, based on the differential expressed genes in resistant cells compared to sensitive cells, were shown. And the top 10 enriched genes were listed. B. ATF6 mRNA expression in sensitive cells or resistant cells under DDP (1.2μg/ml) incubation for 24 hours was measured by qRT-PCR. C. BAD (up), BAX (middle) and BIM (down) mRNA expression in sensitive cells or resistant cells under DDP (1.2μg/ml) incubation for 24 hours was measured by qRT-PCR. D. Expression of CHOP protein level in sensitive and resistant cells was determined by western blotting. E. Expression of CHOP and ATF4 protein level in sensitive cells with ATF4 knockdown for 24 hours and DDP (1.2μg/ml) incubation for another 16 hours was determined by western blotting. F. The level of apoptosis marker C-PARP1 and CHOP in SGC7901 (up) or BGC823 (down) cells with CHOP knockdown and DDP (1.2μg/ml) treatment for 24 hours was detected by western blotting.


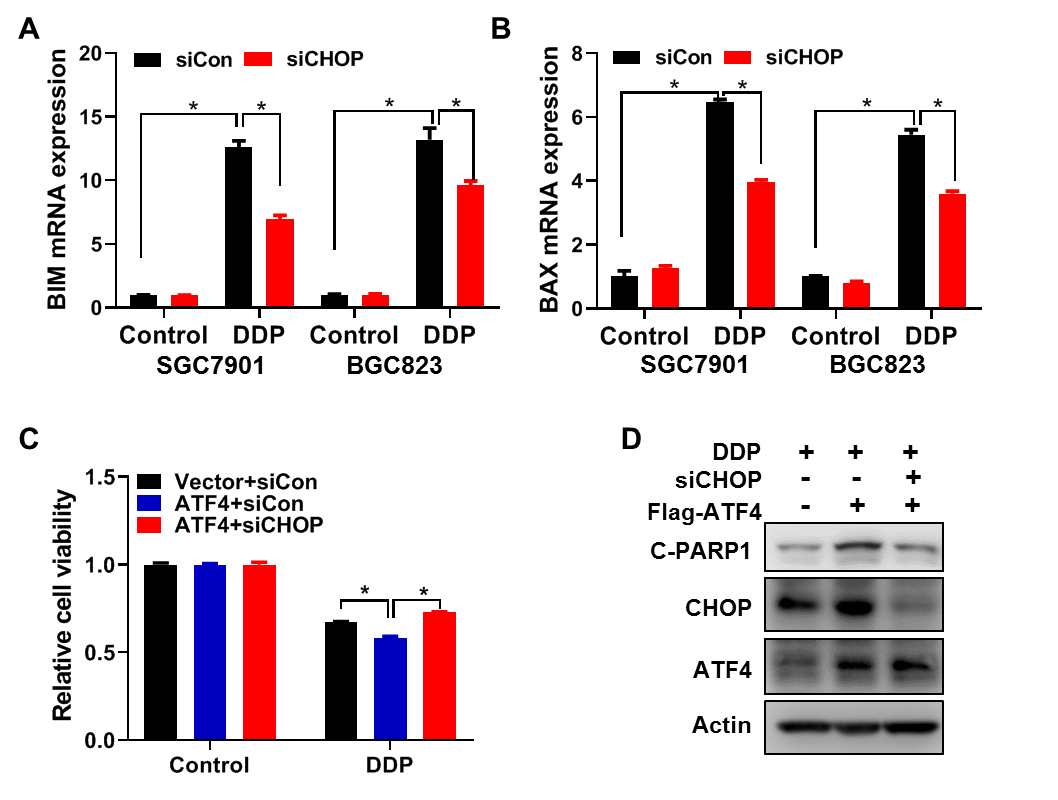


**Supplemental Figure 4. ATF4 stimulates drug-induced apoptosis by activating CHOP transcription**

A and B. Expression of BIM (A) or BAX (B) mRNA level in sensitive cells with CHOP knockdown and DDP (1.2μg/ml) incubation for 24 hours was analyzed by qRT-PCR. C. Cell viability of BGC-R cells with ATF4 overexpression together with CHOP knockdown and DDP treatment as indicated under DDP (6μg/ml) treatment for 24 hours was measured by MTS assay. D. Expression of C-PARP1, CHOP and ATF4 in BGC-R cells with ATF4 overexpression together with CHOP knockdown and DDP treatment as indicated under DDP (6μg/ml) treatment for 24 hours was determined by western blotting.


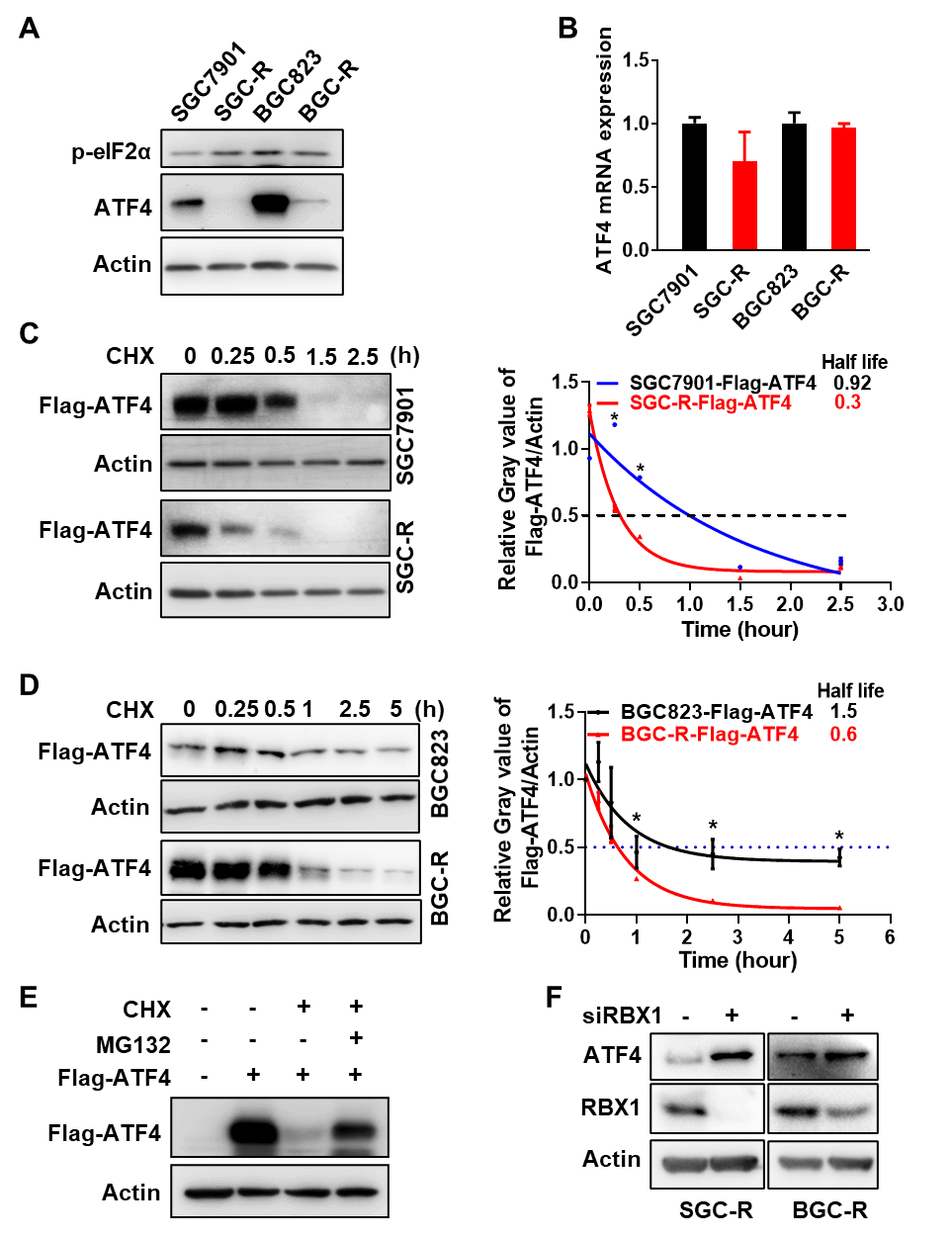


**Supplemental Figure 5. βTrCP enhanced ubiquitination dependent proteasomal degradation of ATF4 in chemo-resistant cells**

A. Expression of p-eIF2α in sensitive and resistant cells was detected by western blotting. B. mRNA expression of ATF4 in sensitive and resistant cells was measured by qRT-PCR. C. Turnover of exogenous Flag-ATF4 in SGC7901 or SGC-R cells under CHX (50μg/ml) incubation was detected by western blotting using anti-Flag antibody. And the relative grey value of Flag-ATF4 compared to Actin was analyzed by Image J, then normalized to the ‘0’ time point sample. The fitted curves were drawn with GraphPad software and the half-life (hours) of Flag-ATF4 turnover was analyzed and shown. D. Turnover of exogenous Flag-ATF4 in BGC823 or BGC-R cells under CHX (50μg/ml) incubation was detected by western blotting using anti-Flag antibody. And the relative grey value of Flag-ATF4 compared to Actin in ‘C’ was analyzed by Image J, then normalized to the ‘0’ time point sample. The fitted curves were drawn with GraphPad software and the half life (hours) of Flag-ATF4 turnover was analyzed and shown. E. Turnover of exogenous Flag-ATF4 in BGC-R cells with or without MG132 (10μM) treatment, and CHX (50μg/ml) incubation was detected by western blotting. F. The expression of ATF4 in SGC-R (left) or BGC-R (right) with RBX1 knockdown was determined by western blotting.


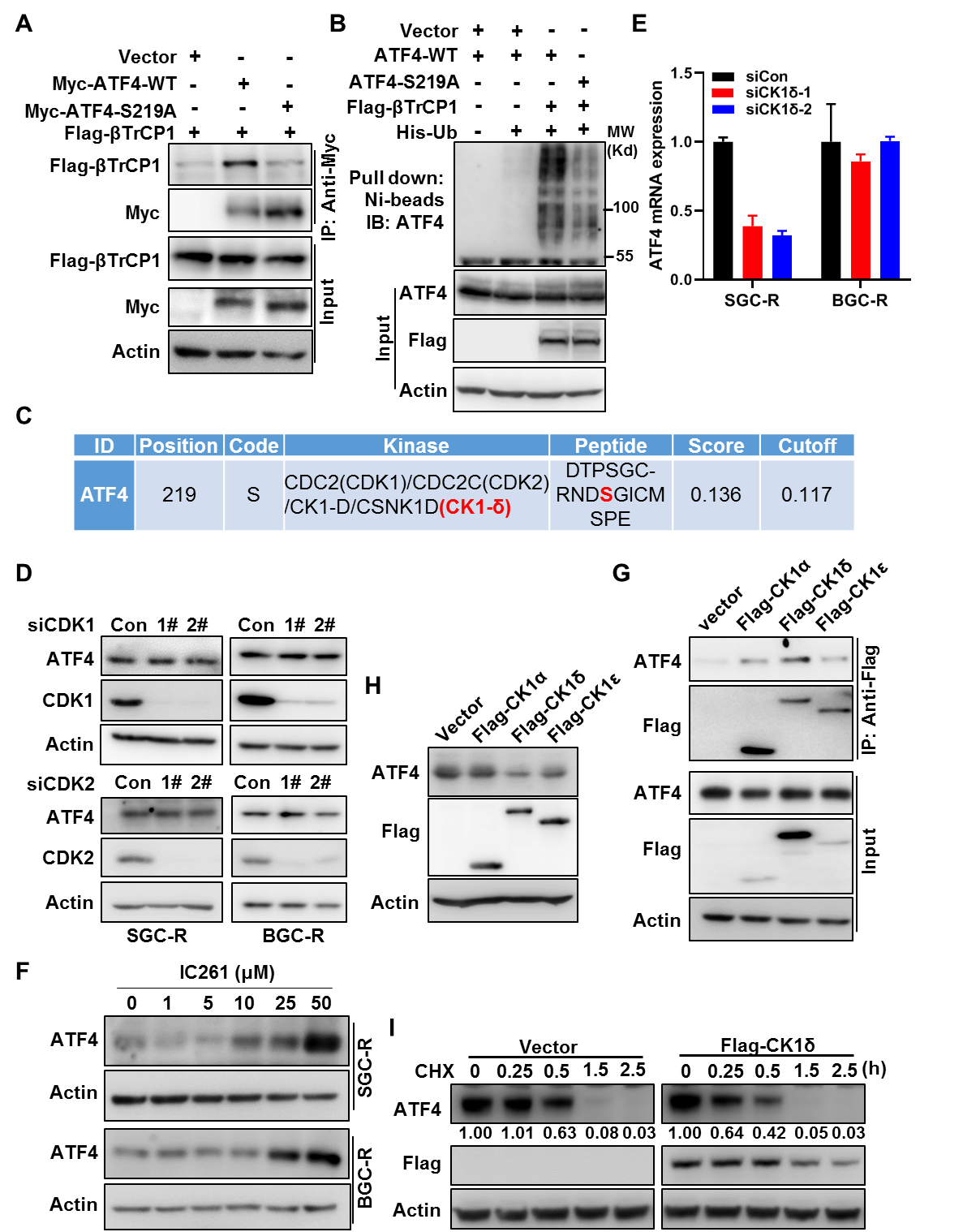


**Supplemental Figure 6. CK1δ phosphorylates ATF4 to stimulate its ubiquitination-dependent proteasomal degradation**

A. Interaction of wild type Myc-ATF4 (Myc-ATF4-WT) or Myc-ATF4-S219A mutant with βTrCP1 in HEK293T cells was determined with anti-Myc co-IP, followed by anti-Flag and anti-Myc immunoblot. B. ATF4-WT or ATF4-S219A ubiquitination level in HEK293T cells with Flag-βTrCP1 overexpression was measured by *in vitro* ubiquitination assay. C. The GPS online software was used to screen the potential kinases for S219 of ATF4. D. Expression of ATF4 in SGC-R or BGC-R cells with CDK1 (up) or CDK2 (down) knockdown was detected by western blotting. E. ATF4 mRNA level in SGC-R or BGC-R with CK1δ knockdown was analyzed by qRT-PCR. F. Expression of ATF4 in SGC-R (up) or BGC-R (down) cells with CK1δ inhibitor IC261 treatment was detected by western blotting. G. Co-IP was performed in HEK293T cells with HA-ATF4 and Flag-CK1α/δ/ε co-transfection with anti-Flag, and the interaction was detected by western blotting with anti-ATF4 and anti-Flag antibodies. H. Expression of ATF4 in BGC823 with exogenous Flag-CK1α/δ/ε overexpression was detected by western blotting with anti-ATF4 antibody, and the expression of Flag-CK1α/δ/ε was confirmed by anti-Flag antibody. I. Under CHX (50μg/ml) treatment, the protein turnover of endogenous ATF4 in SGC7901 cells with or without Flag-CK1δ over-expression was detected by western blotting using anti-ATF4 antibody. The relative grey value of ATF4 compared to Actin was analyzed, and the normalized expression ratio was shown.


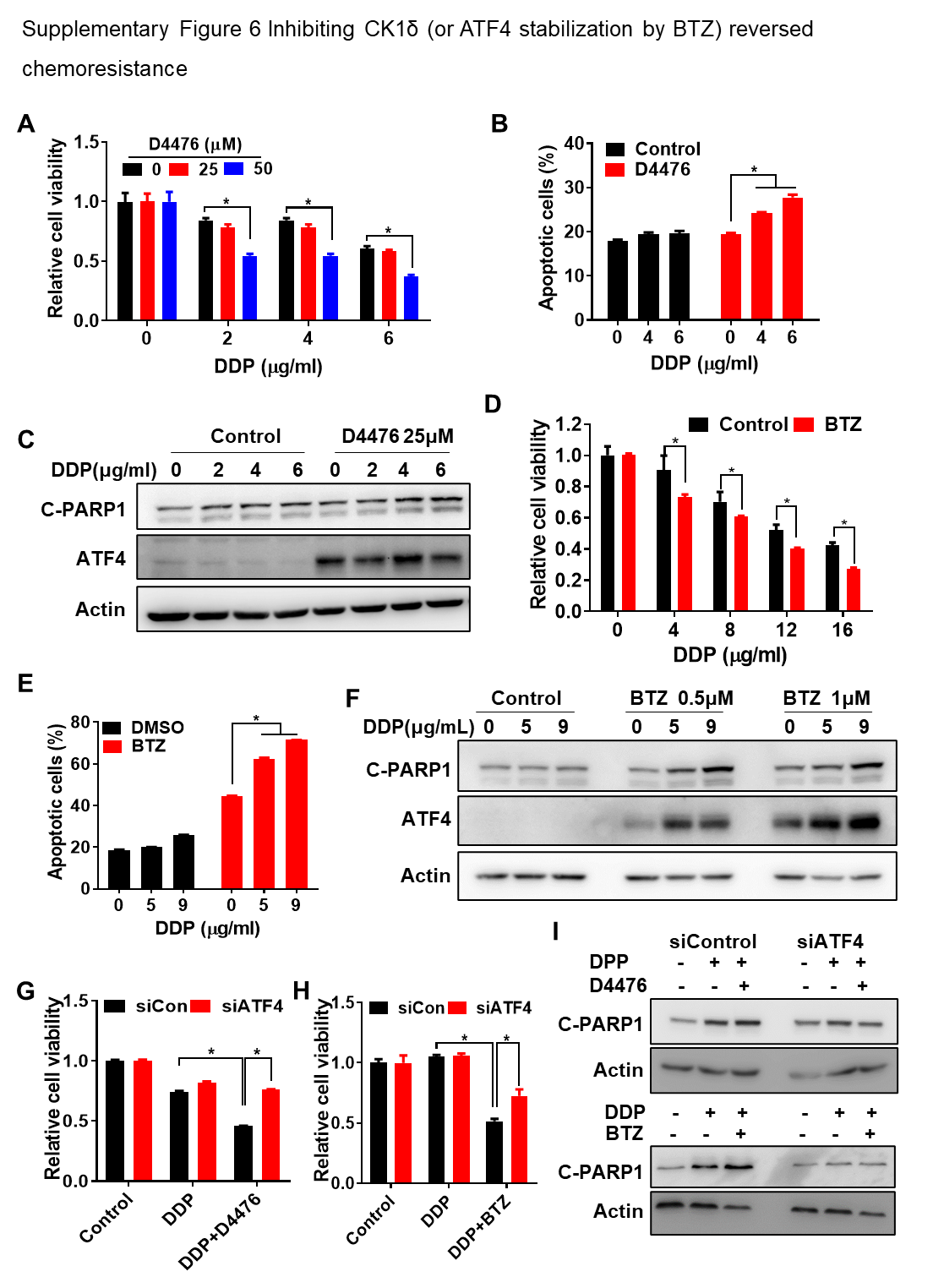


**Supplemental Figure 7. Stabilization of ATF4 protein reverses Chemoresistance.**

A. Cell viability of BGC-R cells with DDP treatment, with/without D4476 (0, 25, 50 μM) combination for 24 hours was measured by MTS assay. B. Apoptosis of BGC-R cells with DDP treatment, with/without D4476 (50 μM) combination for 24 hours was analyzed by PI/Annexin V double staining. C. Expression of apoptosis marker C-PARP1 in BGC-R cells with DDP treatment, with/without D4476 combination for 24 hours was detected by western blotting. Cell viability (D) and apoptosis (E) of BGC-R cells with DDP treatment, with/without BTZ (0.5 μM) combination for 24 hours was measured. F. Expression of apoptosis marker C-PARP1 in BGC-R cells with DDP treatment, with/without BTZ combination for 24 hours was detected by western blotting. Cell viability of BGC-R cells with DDP treatment, with/without D4476 (G) or BTZ (H) combination for 24 hours after ATF4 knockdown was measured by MTS assay. I. Expression of apoptosis marker C-PARP1 in BGC-R cells with DDP treatment, with/without D4476 (25 μM) (up) or BTZ (0.5 μM) (down) combination for 24 hours after ATF4 knockdown was detected by western blotting.


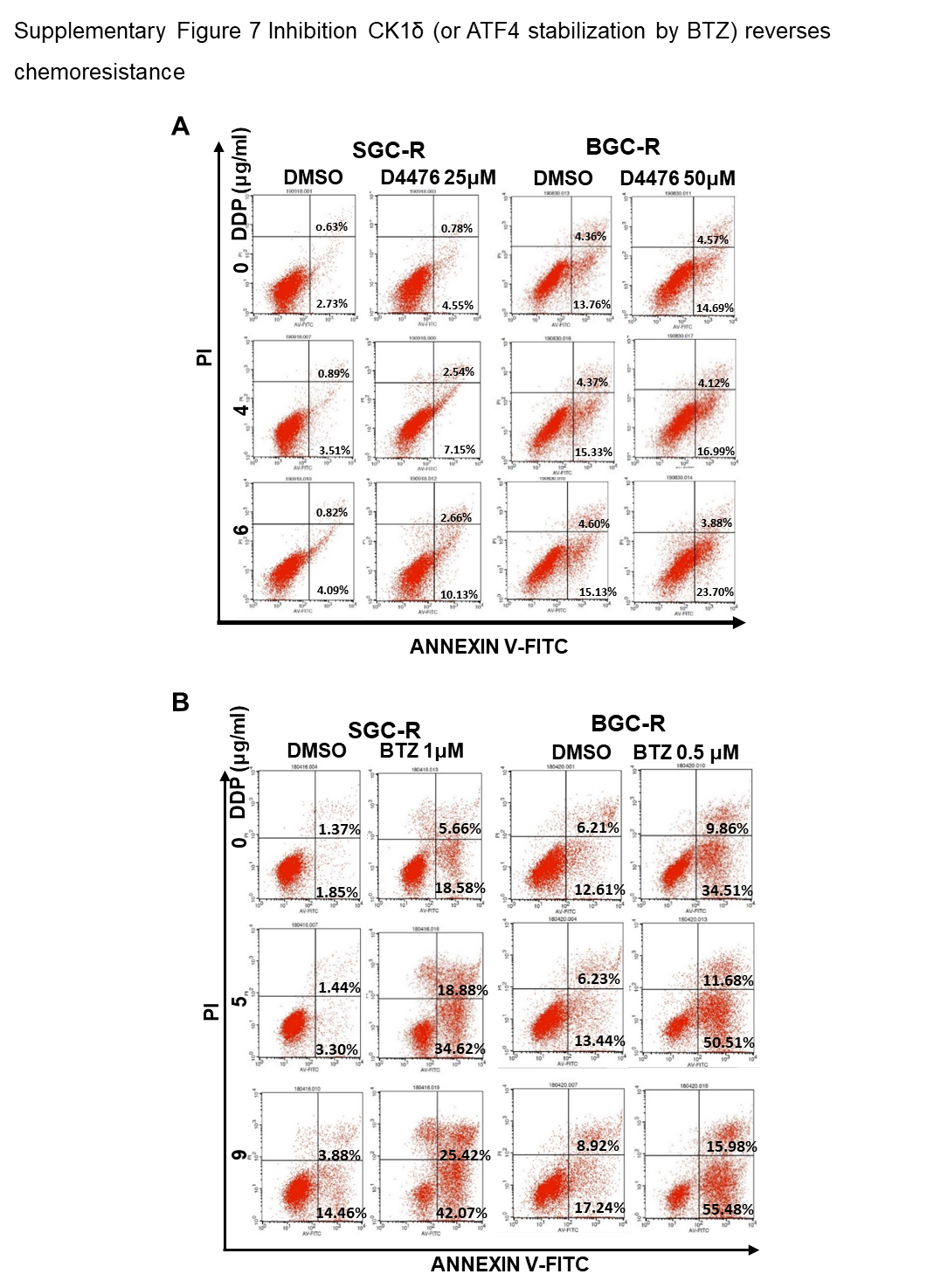


**Supplemental Figure 8. Stabilization of ATF4 protein enhances drug induced apoptosis.**

A. Represent flow cytometric apoptosis pictures of SGC-R (left) or BGC-R (right) cells with DDP treatment, with/without D4476 combination for 24 hours were shown. B. Represent flow cytometric apoptosis pictures of SGC-R (left) or BGC-R (right) cells with DDP treatment, with/without BTZ combination for 24 hours were shown.


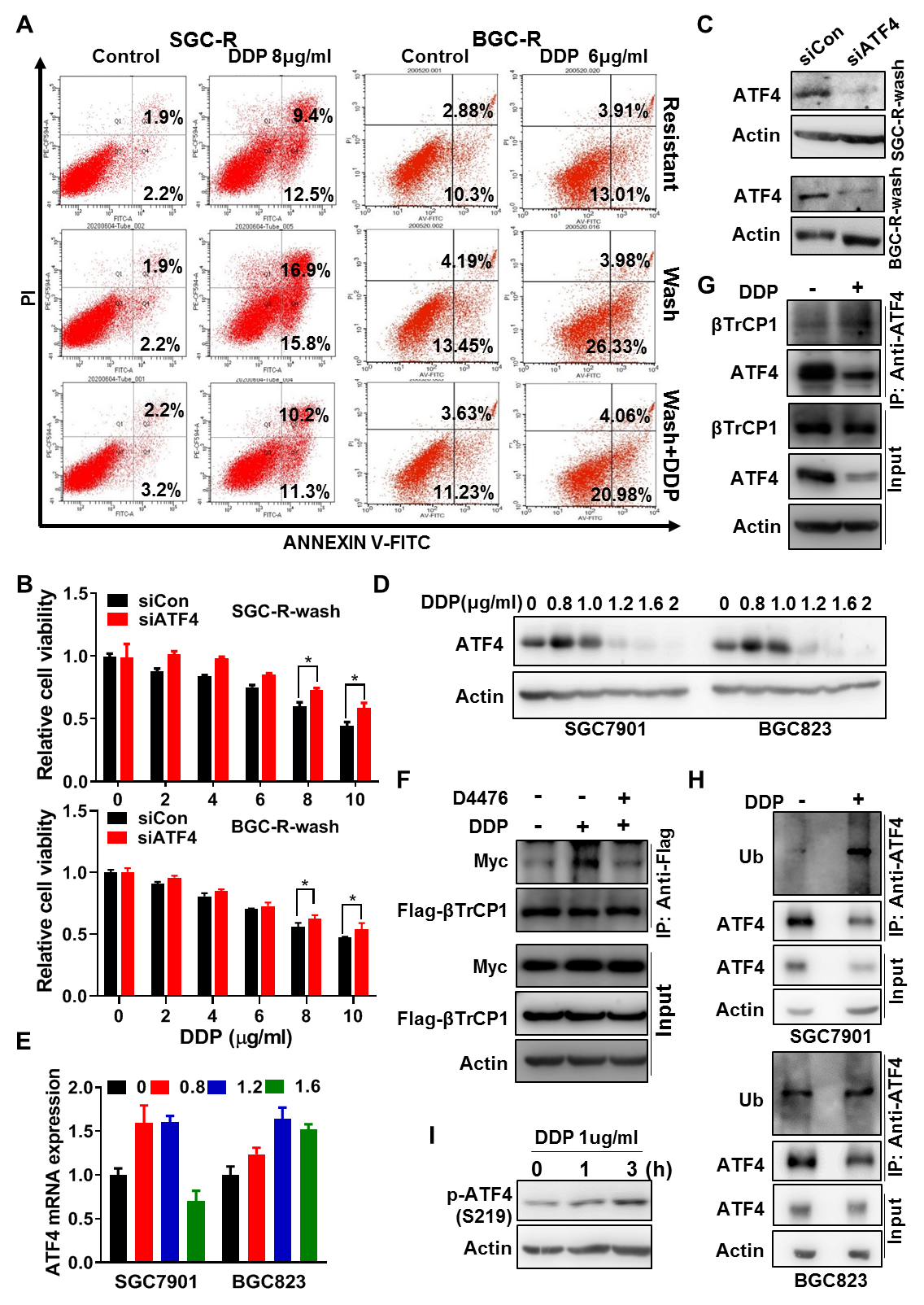


**Supplemental Figure 9. A dynamic ATF4 degradation in adaptive chemotherapy**

A. Represent flow cytometric apoptosis pictures of different resistant cells as indicated under DDP incubation for 24 hours were shown. B. Cell viability of SGC-R-wash (up) or BGC-R-wash (down) with ATF4 knockdown and DDP treatment for 24 hours was measured by MTS assay. C. Knockdown of ATF4 in SGC-R-wash (up) or BGC-R-wash (down) was validated by western blotting. D. ATF4 protein expression in sensitive cells with dose dependent DDP treatment for 24 hours were detected by western blot. E. ATF4 mRNA expression in sensitive cells with dose dependent DDP (0, 0.8, 1.2, 1.6μg/ml respectively) treatment for 24 hours were analyzed by qRT-PCR. F. Interaction of Myc-ATF4 with Flag-βTrCP1 in HEK293T cells with DDP (3μg/ml) and with or without D4476 (25μM) treatment for 12 hours was determined by anti-Flag co-IP, then immunoblot with anti-Myc and anti-Flag. G. Interaction of ATF4 with βTrCP1 in BGC823 cells with/without DDP (1.2μg/ml) treatment for 12 hours was determined by anti-ATF4 co-IP, then immunoblot with anti-βTrCP1 and anti-ATF4. H. ATF4 ubiquitination level in SGC7901 (left) or BGC823 (right) cells with DDP incubation for 12 hours was determined by anti-ATF4 co-IP, and probed with anti-Ub. I. p-ATF4(S219) in BGC823 cells with DDP treatment for 24 hours in different times was detected by western blotting.


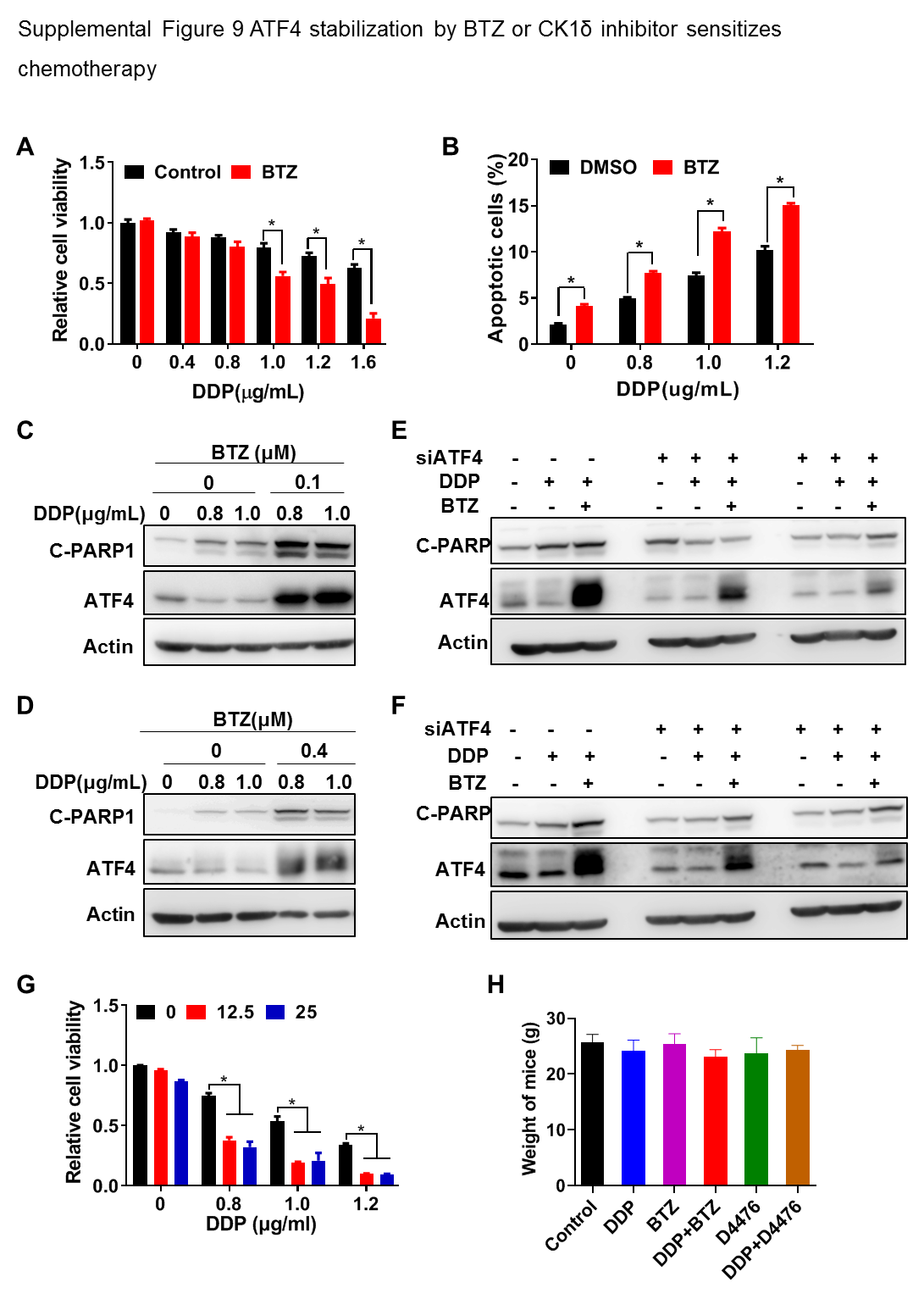


**Supplemental Figure 10. ATF4 stabilization by BTZ or CK1δ inhibitor enhances the efficacy of chemotherapy in gastric cancer**

A and B. Cell viability (A), apoptosis (B) of BGC823 cells with DDP treatment for 24 hours, with/without BTZ (0.4 μM) combination was measured. C and D. Expression of apoptosis marker C-PARP1 in SGC7901 (C) or BGC823 (D) cells with DDP treatment for 24 hours, with/without BTZ combination was detected by western blotting. E and F. After transfection with siATF4s, Expression of C-PARP1 in SGC7901 (E) or BGC823 (F) under DDP incubation for 24 hours, and BTZ (0.1 μM for SGC7901 and 0.4 μM for BGC823) combination or not was determined by western blotting. G. Cell viability of BGC823 with DDP treatment for 24 hours, with/without D4476 (0, 12.5 or 25 μM) combination was measured by MTS assay. H. Mice weight of each group was taken at the end of the experiment.


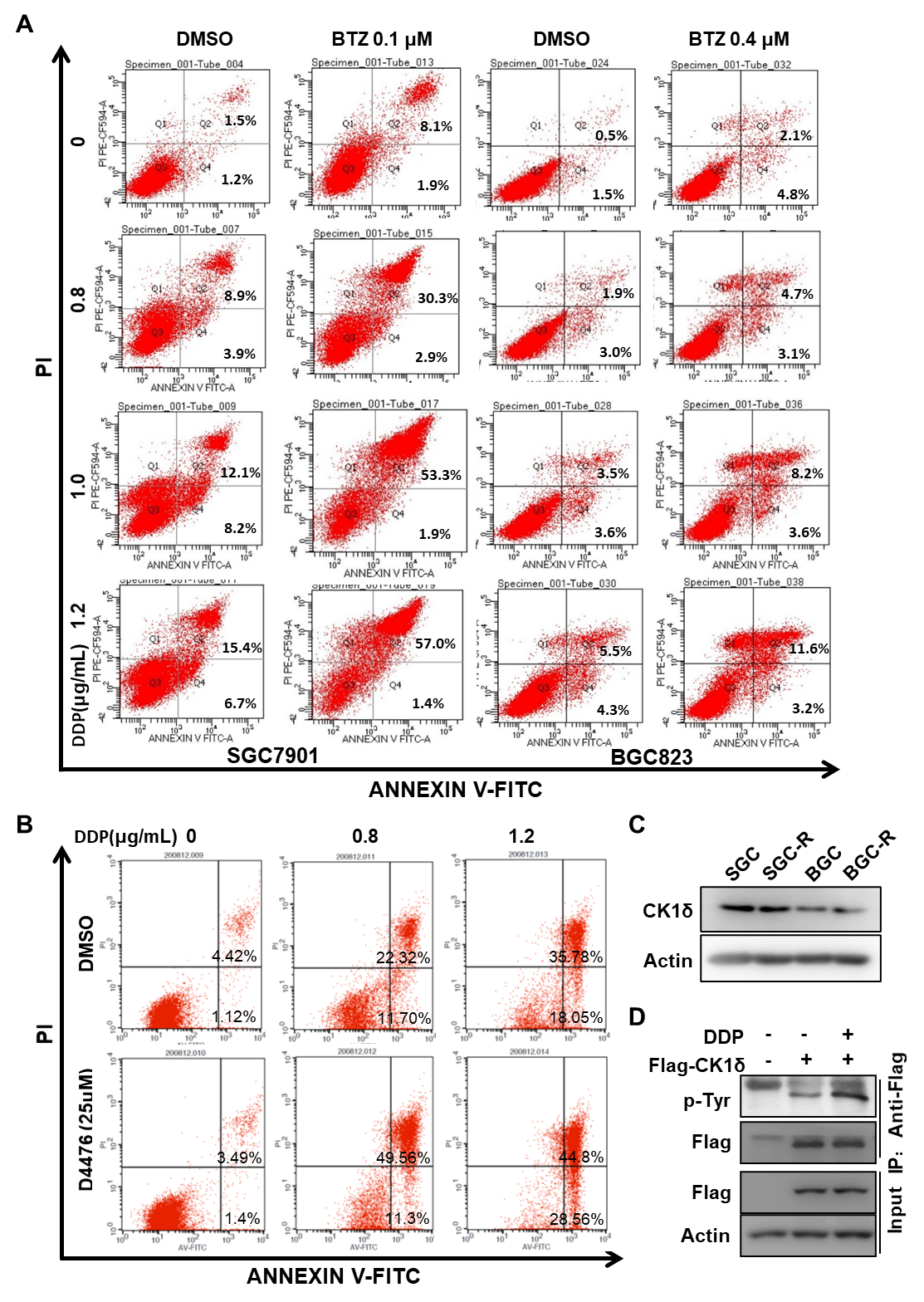


**Supplemental Figure 11. Stabilizing ATF4 enhanced DDP induced apoptosis.**

A. Represent flow cytometric apoptosis pictures of sensitive cells under DDP incubation for 24 hours, with/without BTZ combination were shown. B. Represent flow cytometric apoptosis pictures of BGC823 cells under DDP incubation for 24 hours, with/without D4476 combination were shown. C. Expression of CK1δ in sensitive and resistant cells was detected by western blotting. D. After transfection of Flag-CK1δ, HEK293T cells were treated with DDP for 12 hours, anti-Flag IP was performed and phosphorylation of CK1δ was detected by anti-pan tyrosine antibody (anti-p-Tyr).

**Supplemental Table 1**

| **Primers** | |
| --- | --- |
| **Name** | **Sequence** |
| Actin | F: CACCAACTGGGACGACAT |
|  | R: ACAGCCTGGATAGCAACG |
| ATF4 | F: CTTAAGCCATGGCGCTTCTC |
|  | R: GAAGGCATCCTCCTTGCTGTT |
| CHOP | F: CCTGAAAGCAGACTGATCCAAC |
|  | R: GCAGGGTCAAGAGTGGTGAA |
| ATF6 | F: TCCTCGGTCAGTGGACTCTTA |
|  | R: CTTGGGCTGAATTGAAGGTTTTG |
| ATF4-S219A | F: CTCATACAGATGCCAGCATCATTATCTGAAGGGGTGTCTTC |
|  | R: GAAGACACCCCTTCAGATAATGATGCTGGCATCTGTATGAG |
| CK1δ | F: GACGACAAGCCTGACTACTC |
|  | R: TGTGTGAGGTAGGGGTGAG |
| **siRNAs** | |
| Control siRNA | S: UUCUCCGAACGUGUCACGUTT |
|  | AS: ACGUGACACGUUCGGAGAATT |
| ATF4-1 | S: CUGCUUACGUUGCCAUGAUTT |
|  | AS: AUCAUGGCAACGUAAGCAGTT |
| ATF4-2 | S: CCCUUCAGAUAAUGAUAGUTT |
|  | AS: ACUAUCAUUAUCUGAAGGGTT |
| CHOP-1 | S: GCGCAUGAAGGAGAAAGAATT |
|  | AS: UUCUUUCUCCUUCAUGCGCTT |
| CHOP-2 | S: GCUGAGUCAUUGCCUUUCUTT |
|  | AS: AGAAAGGCAAUGACUCAGCTT |
| CK1δ-1 | S: GGAGACAUCUAUCUCGGUATT |
|  | AS: UACCGAGAUAGAUGUCUCCTT |
| CK1δ-2 | S: GCAACCUGGUGUACAUCAUTT |
|  | AS: AUGAUGUACACCAGGUUGCTT |
| βTrCP | S: GUGGAAUUUGUGGAACAUCTT |
|  | AS: GAUGUUCCACAAAUUCCACTT |
| CDK1-1 | S: GGAUGUGCUUAUGCAGGAUTT |
|  | AS: AUCCUGCAUAAGCACAUCCTT |
| CDK1-2 | S: CCUGGUCAGUACAUGGAUUTT |
|  | AS: AAUCCAUGUACUGACCAGGTT |
| CDK2-1 | S: GUACGGAGUUGUGUACAAATT |
|  | AS: UUUGUACACAACUCCGUACTT |
| CDK2-2 | S: CUCAGAAUCUGCUUAUUAATT |
|  | AS: UUAAUAAGCAGAUUCUGAGTT |
| Culin1 | S: GCUCUACACUCAUGUUUAUTT |
|  | AS: AUAAACAUGAGUGUAGAGCTT |
| RBX1 | S: GGACAACAGAGAGUGGGAATT |
|  | AS: UUCCCACUCUCUGUUGUCCTT |
